# High altitudes, population density, and poverty: Unraveling the complexities of COVID-19 in Peru during the years 2020–2022

**DOI:** 10.1016/j.pmedr.2023.102423

**Published:** 2023-09-15

**Authors:** David A. Vizcardo, Jorge R. Araníbar, César Vladimir Munayco Escate

**Affiliations:** Facultad de Medicina, Universidad Peruana de Ciencias Aplicadas, Lima 15023, Peru

**Keywords:** COVID-19, Peru, Altitude, Population density, Poverty, SARS-CoV-2

## Abstract

**Background:**

Several factors related to hospitalizations, morbidity, and mortality from COVID-19 have been identified. However, limited exploration has been done on geographic and socioeconomic factors that could significantly impact these outcomes.

**Objectives:**

This study aimed to determine whether altitude, population density, and percentage of population in total poverty are associated with COVID-19 incidence per 1000 inhabitants and COVID-19 case-fatality rate in Peru, from 2020 to 2022.

**Methods:**

This study utilized a multiple group ecological design and relied on secondary databases containing daily records of COVID-19 positive cases and deaths due to COVID-19. An epidemiological analysis was performed, subsequently processed using a random effects model.

**Results:**

As of August 2022, Peru had recorded a total of 3,838,028 COVID-19 positive cases and 215,023 deaths due to COVID-19. Our analysis revealed a statistically significant negative association between altitude and COVID-19 incidence (aBETA: −0.004; Standard Error: 0.001; p < 0.05). Moreover, we observed a positive association between population density and incidence (aBETA: 0.006; Standard Error: 0.001; p < 0.05). However, we found no significant association between the percentage of population in total poverty and COVID-19 incidence.

**Conclusion:**

Our study found that an increase in altitude was associated with a decrease in COVID-19 incidence, while an increase in population density was associated with an increase in COVID-19 incidence. High altitude, population density and percentage of population in total poverty does not change case-fatality rate due to COVID-19.

## Highlights

• At higher altitudes, the COVID-19 incidence decreases.
• An increase of population density corresponds to an increase in COVID-19 incidence.
• Percentage of population in total poverty is not associated with COVID-19 incidence.
• Case-fatality rate is not associated with altitude, population density or percentage of population in total poverty.

## 1 Introduction

**Fig. 1:**
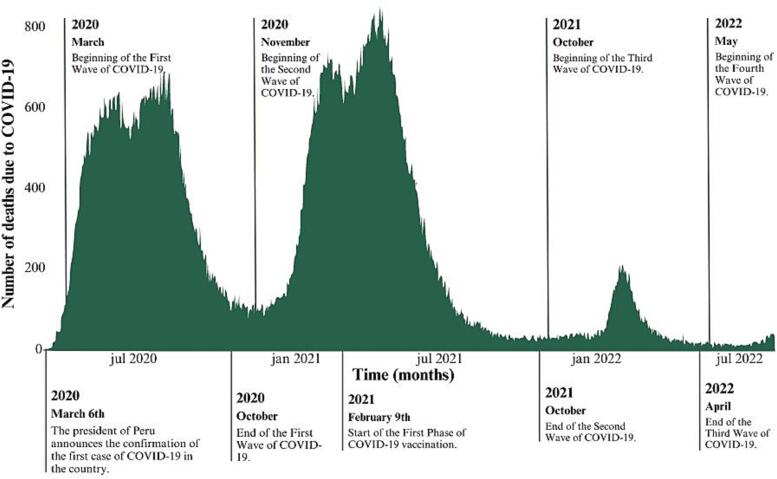
Timeline of events associated with COVID-19 in Peru between 2020 and 2022. Sources: Instituto Nacional de Salud (National Institute of Health) and Centro Nacional de Epidemiología, Prevención y Control de Enfermedades (National Center for Epidemiology, Prevention, and Control of Diseases) – MINSA (Ministry of Health, Peru).

In December 2019, cases of an unidentified pneumonia emerged in Wuhan, China, later identified as COVID-19 (Zheng, 2020). The disease rapidly spread to nearly 20 countries, prompting the World Health Organization to declare it a public health emergency of international concern on January 30, 2020 (Khan et al., 2021). The COVID-19 pandemic has caused thousands of deaths and millions of infections, with Latin American countries, which have fragile economies and weak health systems, being the most affected, with high numbers of deaths caused by SARS-CoV-2, This encompassed Peru, which by June 2021 was reported to have approximately 150% more COVID-19-related deaths than anticipated, compounded by additional challenges such as the deteriorating state of the Peruvian healthcare system (Ashktorab et al., 2021, Covid, 2020). The chronology of COVID-19 in Peru is depicted in Fig. 1. Consequently, extensive research is being conducted on the clinical, pathogenic, and epidemiological aspects of the virus to achieve a comprehensive understanding of COVID-19 (Tsai et al., 2021).

Several factors have been identified as associated with higher rates of hospitalizations, morbidity, and mortality in individuals with COVID-19. Studies have consistently shown that older individuals and males are more vulnerable to the disease (Haitao et al., 2020, Salimi and Hamlyn, 2020). Additionally, the presence of chronic conditions like diabetes, obesity, and hypertension can worsen the prognosis and outcome of COVID-19 (Seclén et al., 2020, Ejaz et al., 2020 Dec). However, other factors such as geographic, demographic, and socioeconomic factors have not been thoroughly explored (Millet et al., 2020, Patel et al., 2020 Jun, Krumer-Nevo and Refaeli, Pun et al., 2020 Sep 1).

In Peru and other countries like Bolivia, Ecuador, Colombia, and Brazil, research suggests that altitude may serve as a protective factor against COVID-19 morbidity and mortality (Thomson et al., 2021 Jun 1, Fernandes et al., 2021, Arias-Reyes et al., 2021). However, the protective effect of altitude on COVID-19 remains uncertain, as previous studies on influenza and recent research on SARS-CoV-2 did not find a positive association and even reported a harmful effect of altitude. Moreover, several other factors can influence the proposed hypothesis, and the extent of their impact is yet to be fully understood (Pun et al., 2020 Sep 1, Pérez-Padilla et al., 2013, Segovia-Juarez et al., 2020).

Regarding population density, it is noteworthy that SARS-CoV-2 transmission primarily occurs via aerosols and droplets generated from coughing and sneezing (Salian et al., 2021). Therefore, in March 2020, the World Health Organization (WHO) emphasized that social isolation was a critical pillar in controlling the incidence of COVID-19 infections (WHO, 2021). As a result, numerous studies have examined the relationship between population density and COVID-19 infections and fatalities, yielding varied findings (Pascoal and Rocha, 2022, Bhadra et al., 2021, Ilardi et al., 2021, Wong and Li, 2020, Diao et al., 2021).

**Fig. 2:**
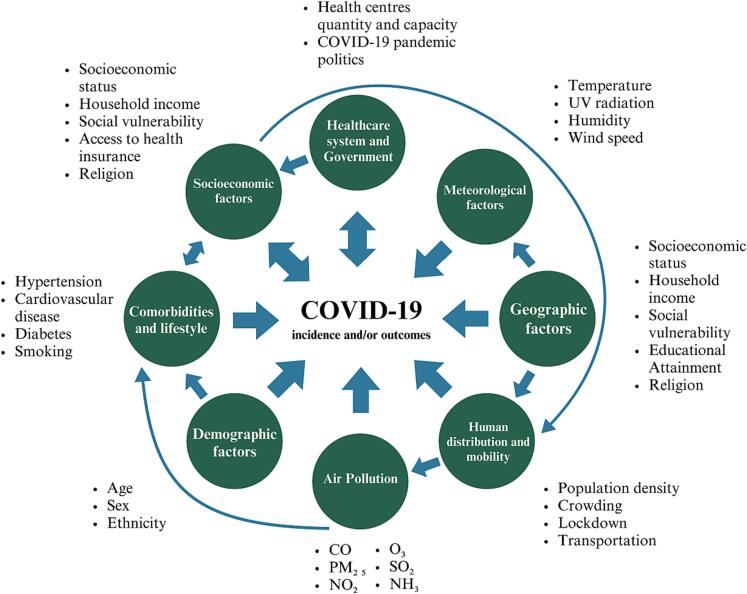
Factors that might influence COVID-19 incidence and/or outcomes. Mono-directional and bi-directional arrows indicate the flux of influence of the factors and outcomes. Adapted from Han J, Yin J, Wu X, Wang D, Li C. Environment and COVID-19 incidence: A critical review. J Environ Sci (China) [Internet]. 2023 [cited 23 Aug 10], 124:933–51.

Regarding socio-economic factors, Patel et al. contend that the assertion that “COVID-19 does not discriminate” is impractical since it overlooks the specific vulnerability of lower socio-economic strata to infection and adverse health outcomes (Patel et al., 2020). This susceptibility is attributable to a range of factors, including overcrowding, mandatory in-person work, increased reliance on public transportation, limited access to health services, higher prevalence of comorbidities, among others (Covid, 2020, Patel et al., 2020 Jun, Krumer-Nevo and Refaeli, Chang et al., 2021, Institute of Health Equity [Internet]. [cited, 2021). Fig. 2 summarizes these and other factors found in the international literature that could influence COVID-19 incidence and/or outcomes (Han et al., 2023, Liu et al., 2023).

Peru, spanning an area of 1,285,215.6 km2 and boasting an altitude exceeding 4,700 m above sea level, is a megadiverse country, as “the verticality imposed by the Andean mountain range (Peru), near the Pacific Ocean, configures a complex and varied climatic dynamic, generating high biological diversity, ecosystems, and production zones” (MINAM-ESDA-2013) (del Perú and Nacional, 2021, del Perú, 2023). The intricate combination of climates, soils, and microenvironments leads to variations in the physiology of people and their environment (del Ambiente, 2021). Furthermore, during the peak of the COVID-19 pandemic, disparities across different geographical, social, and economic groups became evident, thus warranting an investigation of how these variables act on specific populations. Accordingly, this study aims to examine the altitude, population density, and percentage of population in total poverty as potential factors associated with COVID-19 incidence and case fatality rate (CFR), to enhance our understanding of the epidemiology of COVID-19 and facilitate the development of future prevention and control measures against this disease not only in Peru but also in other countries (de Salud, 2021).

## 2 Materials and Methods

### 2.1 Design and population

This study adopts a multiple-group ecological analytical design, using secondary daily record databases for COVID-19 positive cases (CPC) and deaths due to COVID-19 (DC) which were later transformed into incidence per 1000 inhabitants and case-fatality rate. The study population consists of the 196 provinces of Peru during the period of 2020–2022, with an approximate population of 32 million inhabitants for 2020 (Ministry of Health and National, 2022). Given the nature of this study, no sampling or sample calculation was performed. Therefore, all elements of the databases within the determined period were utilized.

### 2.2 Procedures

For this study, we collected data on CPC and DC from all 196 provinces in Peru. To accomplish this, we utilized secondary databases containing daily records of both CPC and DC, and calculated the COVID-19 incidence and CFR. These databases were procured from the National Institute of Health and the National Center for Epidemiology, Prevention, and Control of Diseases (Peru), both of which are accessible through the National Platform of Open Data of the Government of Peru (del Perú and Nacional, 2023). Additionally, covariates such as altitude, population density, and the percentage of the population in total poverty were obtained from the National Center for Strategic Planning (Peru) (del Perú and Nacional, 2021). The data were sorted by province and week between 2020 and 2022 using Microsoft Excel 2013, resulting in a final database.

### 2.3 Data analysis

To determine the independent variables that explain COVID-19 incidence and case fatality rate, we performed a panel data analysis, which allows the analysis of the same entities over time, using a random effects regression model with province and time (in months) classification. To validate this model, we conducted diagnostic tests for heteroscedasticity, serial correlation (Lagrange Multiplier test [ML]), and cross-sectional dependence (Breusch-Pagan test).

We compared model fit using the adjusted r-squared (r2). The final model's statistical significance was defined as the minimum model containing only variables that were significant (p < 0.05) in two-tailed Z-tests, had a higher r2, and were consistent with the theoretical framework. We utilized RStudio® software to conduct all analyses, manage the data, and determine the regression model's random effects.

### 2.4 Ethical considerations

Ethical approval for the study protocol was obtained from the Institutional Ethics Committee of the Universidad Peruana de Ciencias Aplicadas (approval code PI 734–21). The use of secondary databases ensured the confidentiality and privacy of individual data, as the information provided was anonymous and did not contain any identifiers.

## 3 Results

In Peru, the number of CPC recorded until August 2022 was over 4 million. However, the data was filtered to remove more than 190,000 records that were incomplete in terms of data regarding the province where the case was detected or the date of detection, resulting in a final sample of 3,838,028 CPC. Similarly, DC recorded until August 2022 was 215,028. After filtering out five records that did not indicate the province of death, a final sample of 215,023 DC was obtained.

To analyze the variation of epidemiological changes at different altitudes, the Amazonas department was selected as a model due to its diverse range of altitudes, ranging from 189 m above sea level (Condorcanqui) to 2359 m above sea level (Luya). For a more comprehensive understanding of the epidemiological patterns observed in each department, refer to the appendix section, where detailed information, including data and graphics, from other departments can be found.

Regarding the bivariate analysis, we present 3D diagrams to facilitate a comprehensive observation of the presence or absence of patterns between the frequencies of CPC and DC regarding the analyzed independent variable. The temporal axis, “X,” represents time in months. The spatial axis, “Y,” denotes each province within the scrutinized department, arranged from lowest to highest according to the independent variable. Finally, the frequency of CPC or DC is represented by the “Z” axis.

**Fig. 3:**
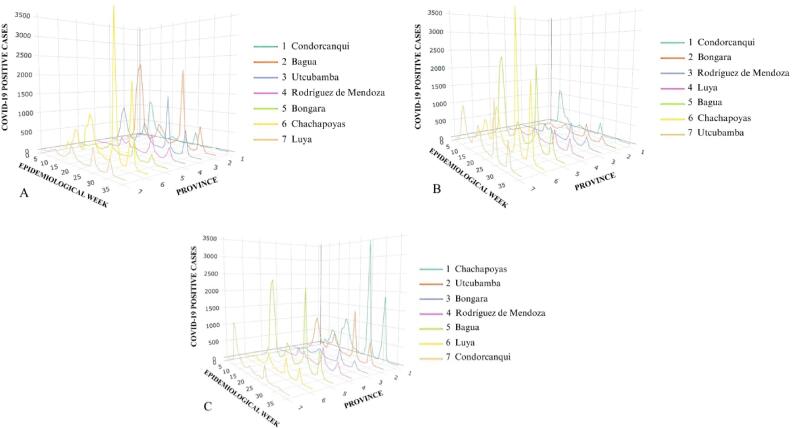
COVID-19 positive cases according to altitude (A), population density (B) and percentage of population in total poverty (C) in Amazonas in the period 2020–2022.

In reference to the CPC, as illustrated in Fig. 3, Luya, the province with the highest altitude in Amazonas, has one of the lowest frequencies of CPC. On the other hand, Chachapoyas, the second-highest province, has one of the highest frequencies of CPC. Therefore, no apparent pattern emerges regarding the altitude of the provinces and their frequency of CPC in the case of Amazonas. Conversely, when examining the same variable in other departments, a discernible pattern emerges wherein higher altitudes correspond to lower frequencies of CPC, although this is not uniformly consistent (see Appendix 3).

It is also shown that, in Amazonas, as the population density of its provinces increases, the frequencies of CPC tend to be higher, a pattern that is repeated in most departments (see Appendix 4). With regards to the percentage of population in total poverty (PPT), it is not revealed any apparent pattern between PPT and the frequencies of CPC in the provinces of Amazonas. Nevertheless, upon examining the other departments, it becomes evident that in some of them, a pattern emerges whereby a lower percentage of total poverty corresponds to a higher frequency of CPC (see Appendix 5).

**Fig. 4:**
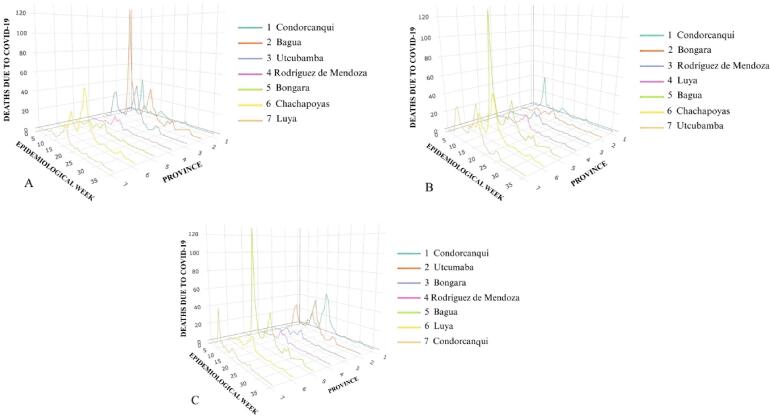
Deaths due to COVID-19 according to altitude (A), population density (B) and percentage of population in total poverty (C) in Amazonas in the period 2020–2022.

In terms of DC, Fig. 4 illustrates that in Amazonas, Bagua, the second-lowest altitude province, has the highest frequency of DC, followed by Chachapoyas with the second-highest frequency. Thus, in the department of Amazonas, there appears to be no clear association between altitude and the frequency of DC in its provinces. Conversely, upon examining the same variable in other departments, although this is not universally consistent (see Appendix 6). It is also demonstrated that in Amazonas provinces with lower population densities tend to have lower frequencies of DC, which increase in proportion to population density. This pattern is consistent across most departments, although Pasco and Moquegua present unique patterns in which provinces with lower population densities have higher numbers of DC compared to their higher density counterparts (see Appendix 7). With regards to the relationship between PPT and the frequencies of DC in the provinces of Amazonas, any discernible pattern is appreciated. However, upon examining the other departments, an association becomes evident in which lower PPT corresponds to higher frequencies of DC (see Appendix 8).

Multivariate analysis was performed and the results are presented in Table 1, Table 2. A random-effects model was utilized to examine the relationship between the COVID-19 incidence or CFR due to COVID-19 and the independent variables. The analysis of COVID-19 incidence (Table 1) revealed that an increase of one unit in altitude was associated with a decrease in COVID-19 incidence of 0.004 (aBETA: −0.004; standard error: 0.001; p-value < 0.05), whereas an increase of one unit in population density was associated with an increase in COVID-19 incidence of 0.006 (aBETA: 0.006; standard error: 0.001; p-value < 0.05). However, the analysis of the case fatality rate due to COVID-19 (Table 2) indicated that there is no significant association with altitude, population density or percentage of population in total poverty.

**Table 1:** Crude and adjusted random effects model of COVID-19 incidence.

Variables	BETAc	Standard error	p-value	BETAa	Standard error	p-value
Altitude ^†^	−0.003	0.001	<0.001	−0.004	0.001	<0.001
Population density ^††^	0.003	0.001	0.015	0.006	0.001	<0.001
Percentage of population in total poverty*	−0.745	0.046	<0.001			

(**^†^**) Variable adjusted by population density.

(**^††^**) Variable adjusted by altitude.

(*) Variable removed from the adjusted analysis due to becoming non-significant (p > 0.05).

**Table 2:** Crude random effects model of case fatality rate due to COVID-19.

Variables	BETAc	Standard error	p-value
Altitude	0.00	0.00	0.797
Population density	0.00	0.01	0.661
Percentage of population in total poverty	0.11	0.24	0.647

## 4 Discussion

The COVID-19 pandemic has led to a health, social, and economic crisis, and several hypotheses have been proposed regarding the behavior of the new disease (Anser et al., 2021, Xiang et al., 2021). In the search for a better understanding of the epidemiological behavior of SARS-CoV-2, the observation of a possible association between geographic altitude and COVID-19 infection has emerged, leading to several studies exploring this relationship. Thomson et al. conducted a study in Peru and concluded that altitude is a protective factor against COVID-19 mortality in populations above 2500 m.a.s.l. (Thomson et al., 2021). Similarly, Arias et al. analyzed data from 23 countries in the Americas and found that the incidence of COVID-19 decreased significantly from 1,000 m.a.s.l (Arias-Reyes et al., 2021). Another study conducted in Brazil found that the relative incidence and mortality rate were lower in higher-altitude cities (Fernandes et al., 2021). Our results also showed a negative association between the altitude of the provinces in Peru and the COVID-19 incidence per 1000 inhabitants. However, it is essential to note that no such association was observed with the case-fatality rate.

Several hypotheses have been proposed to explain this association, such as adaptation to chronic hypoxia at high altitudes, decreased expression of ACE2, a transmembrane protein important for SARS-CoV-2 infection, and the involvement of Hypoxia-Inducible Factor (HIF) and erythropoietin (EPO). These hypotheses could explain a lower susceptibility to infection and a more favorable disease progression in inhabitants of high-altitude regions (Arias-Reyes et al., 2020, Choquenaira-Quispe et al., 2020, Afsar et al., 2020, Soliz et al., 2020).

However, our findings are not entirely consistent with previous studies. For instance, a study conducted in Mexico that investigated cases of respiratory illness during the early stages of the H1N1 influenza pandemic found a positive association between higher altitude and increased hospitalization and mortality rates, even after adjusting for potential confounders (Salimi and Hamlyn, 2020). Similarly, a study in Peru reported that fatality rates were not influenced by altitude and that, although the number of deaths decreased at higher altitudes, a higher proportion of deaths occurred among females (Segovia-Juarez et al., 2020). Furthermore, Pun et al. have pointed out that various environmental, political, temporal, and health-related factors may act as confounding variables when evaluating altitude as a protective factor against COVID-19 (Pun et al., 2020 Sep 1). These factors include but are not limited to, UV radiation, humidity, temperature, pollution, lifestyle, population density, and socioeconomic status (Liu et al., 2023, Burtscher et al., 2020). Our study did not account for all these factors, and their exclusion may have impacted our results.

In addition to the COVID-19 prevention recommendations issued by the World Health Organization (WHO), the potential impact of population density on the transmission of SARS-CoV-2 has been investigated. For example, Bhadra, Mukherjee, and Sarkar conducted a study in India that found a moderate association between case positivity rate (South and Post, 2021). Similarly, Illardi et al. reported a significant positive association between COVID-19 positive cases, deaths, and CFR in Italy, suggesting that population density may be a crucial factor in determining morbidity in that country (Ilardi et al., 2021). Although our results align with prior research concerning COVID-19 incidence patterns but not CFR, the association between population density and COVID-19 transmission may not be as straightforward as it seems. Diao et al. observed in China that population density did not significantly affect the duration of COVID-19 transmission and decline, despite China having a high population density (Diao et al., 2021). Nevertheless, this phenomenon could be explained by the strict containment measures implemented in China (South and Post, 2021).

Therefore, it is possible that population density may not be a significant risk factor for COVID-19 transmission, as it does not necessarily imply clustering or overcrowding, which have been identified as risk factors (Mitlin, 2020). Moreover, the high COVID-19 incidence in areas with high population density may be attributed to other factors, such as poverty and inadequate health infrastructure (Martins-Filho, 2021). Thus, our findings may reflect factors that are associated with population density, rather than population density itself, leading to a spurious association (Ramírez-Valverde et al., 2011). To address this limitation, we included the percentage of the total poverty indicator in our model. However, this indicator may not capture all aspects of poverty that could contribute to COVID-19 incidence and CFR due to COVID-19 (Patel et al., 2020).

Finally, although our study did not find an association between COVID-19 incidence or CFR and the percentage of total poverty after adjusting the model, other studies have reported different findings. For example, Adhikari et al. reported a higher risk of COVID-19 infections and deaths in counties with higher poverty in the United States, regardless of ethnic diversity (Adhikari et al., 2020). Similarly, Jung J. and Shrestha V. found that poor neighborhoods in the United States had the highest number of COVID-19 cases, primarily due to the lack of resources for effective isolation (Jung et al., 2021).

## 5 Limitations

When interpreting our results, it is important to consider the limitations of this study. First, there is the possibility of spurious associations, as the independent variables may not have a true significant effect on the outcome. Second, we acknowledge the presence of the ecological fallacy, whereby group-level results are applied to generate associations at the individual level (Diez Roux, 2008). Third, ecological studies are inherently prone to biases, such as confounding bias, where it may be difficult to adjust for other factors that influence the outcome, as well as bias stemming from geographic variability within the unit of analysis. Moreover, the use of secondary databases poses the risk of data entry errors or processing incorrect data, which can affect the accuracy and fidelity of the data.

To mitigate these limitations, we suggest conducting similar studies in countries or regions with similar characteristics, to allow for more robust comparative analyses. Despite these limitations, our study represents one of the first in Peru to examine the geographic, demographic, and socioeconomic factors associated with COVID-19. As such, our findings can serve as a basis for guiding the development and implementation of guidelines and strategies aimed at mitigating the spread of the virus in the country.

## 6 Conclusions

The hypothesis that altitude and other geographic factors play a role in COVID-19 transmission has raised interest in the scientific community. Our study found a negative association between altitude and COVID-19 incidence and a positive association between population density and COVID-19 incidence, but no association. with poverty. Our study contributes to understanding the transmission dynamics of COVID-19 by identifying that large cities with high-density populations fuel the transmission of airborne diseases like COVID-19. Further, the negative association with altitude explains the distribution of large cities in Peru, which are located mainly in the capital of the departments in the coastal region. Additionally, population density is low at high altitudes.

This study highlights that public health interventions must focus on large cities during the pandemic because dynamic transmission of any airborne disease persists in cities larger than 200,000 inhabitants. High-density populations facilitate infectious disease transmission because of the large number of contact and social networks. On the other hand, poverty could ignite not only transmission but adverse outcomes. Our study did not show any association between poverty and the case fatality rate, probably because of Peru's limited health system response during the COVID-19 pandemic.

## Funding

This research did not receive any specific grant from funding agencies in the public, commercial, or not-for-profit sectors.

## CRediT authorship contribution statement

**David A. Vizcardo:** Conceptualization, Methodology, Validation, Formal analysis, Data curation, Writing – original draft, Writing – review & editing, Visualization. **Jorge Rodríguez Araníbar:** Conceptualization, Methodology, Validation, Formal analysis, Data curation, Writing – original draft, Writing – review & editing, Visualization. **César Vladimir Munayco Escate:** Conceptualization, Methodology, Software, Validation, Formal analysis, Data curation, Writing – review & editing.

## Declaration of Competing Interest

The authors declare that they have no known competing financial interests or personal relationships that could have appeared to influence the work reported in this paper.

## Data Availability

Data will be made available on request.
