# Supplementary material for: High altitudes, population density, and poverty: Unraveling the complexities of COVID-19 in Peru during the years 2020–2022
Source: Prev Med Rep. 2023 Sep 15;36:102423. doi: 10.1016/j.pmedr.2023.102423 (PMC10518345; doi:10.1016/j.pmedr.2023.102423)
Supplement: Supplementary data 1 [file mmc1.docx]

**APPENDIXES**

1. **COVID-19 POSITIVE CASES BY DEPARTMENT DURING THE PERIOD 2020-2022.**​​

| 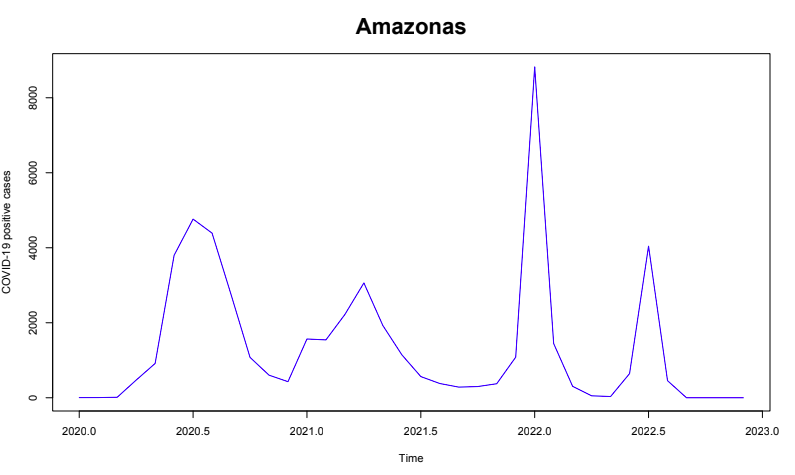 | 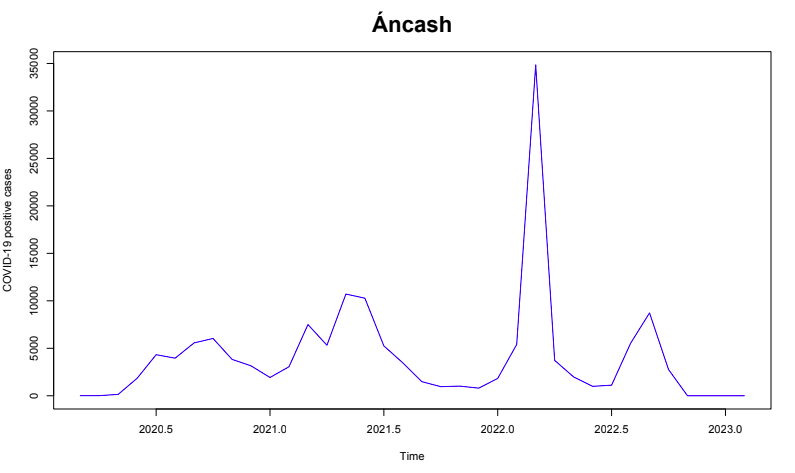 |
| --- | --- |
| 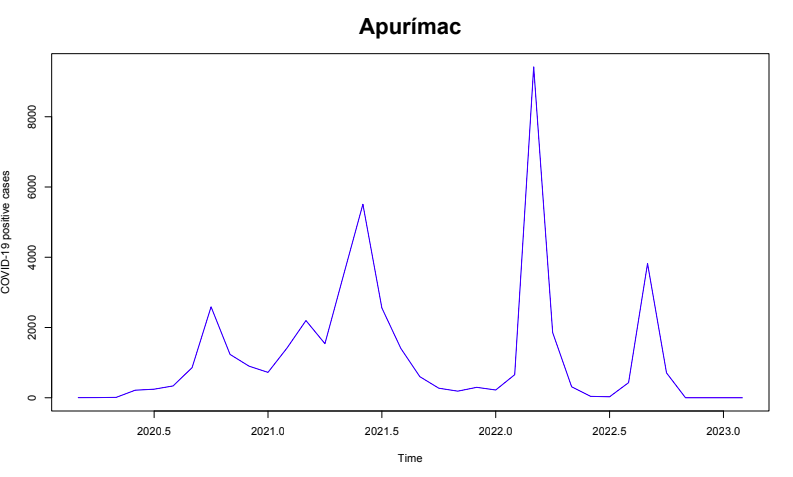 | 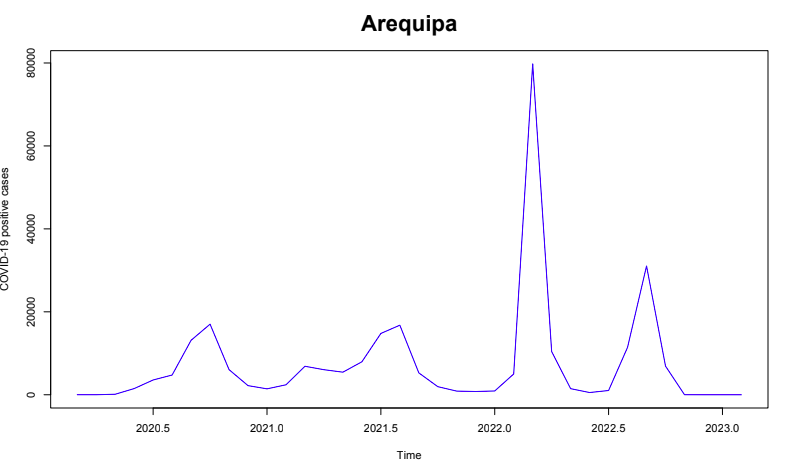 |
| 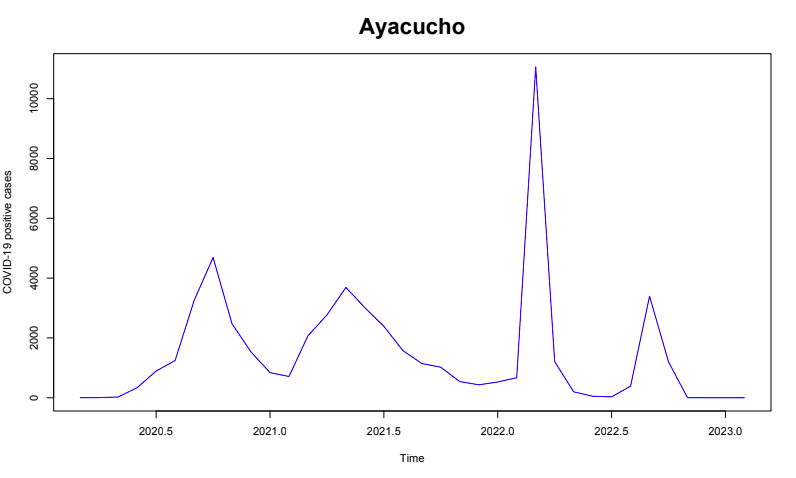 | 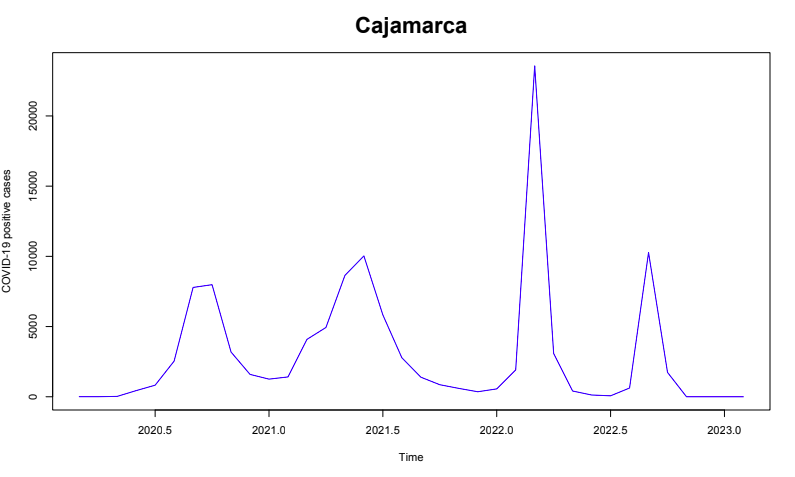 |
| 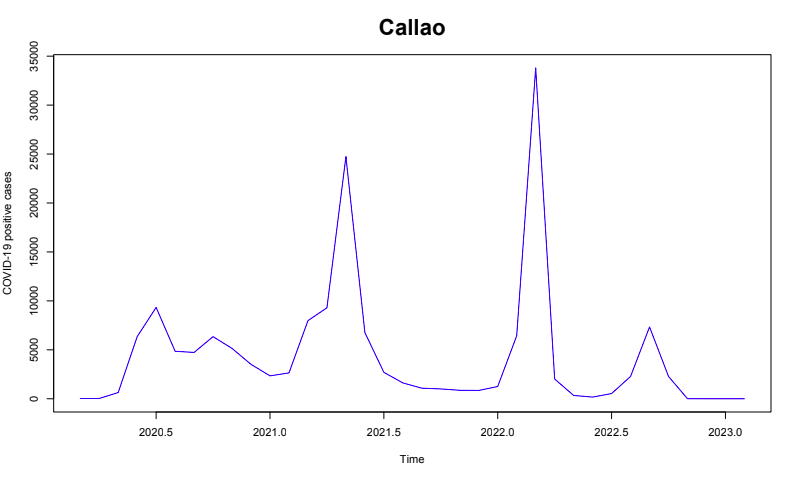 | 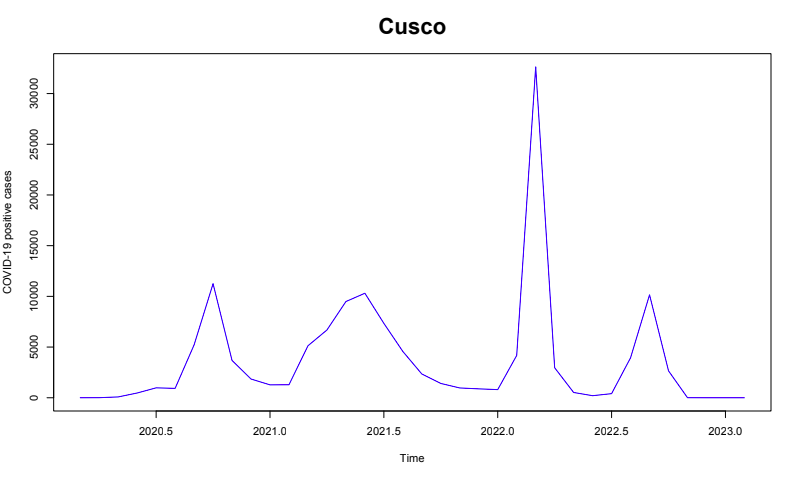 |
| 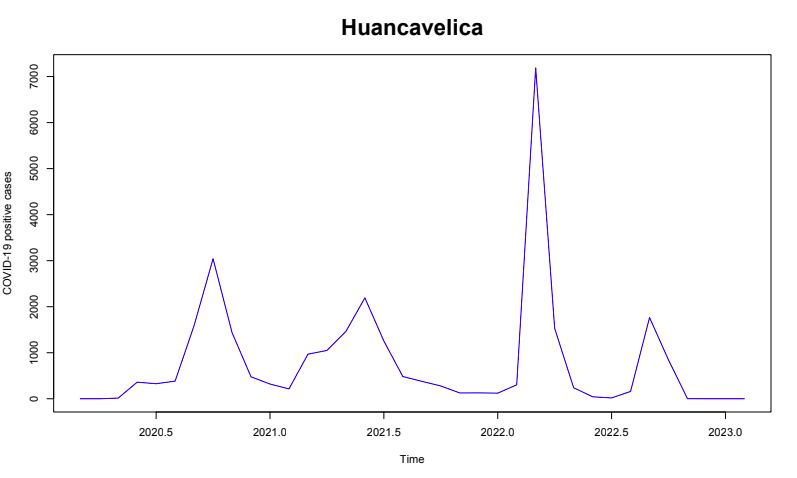 | 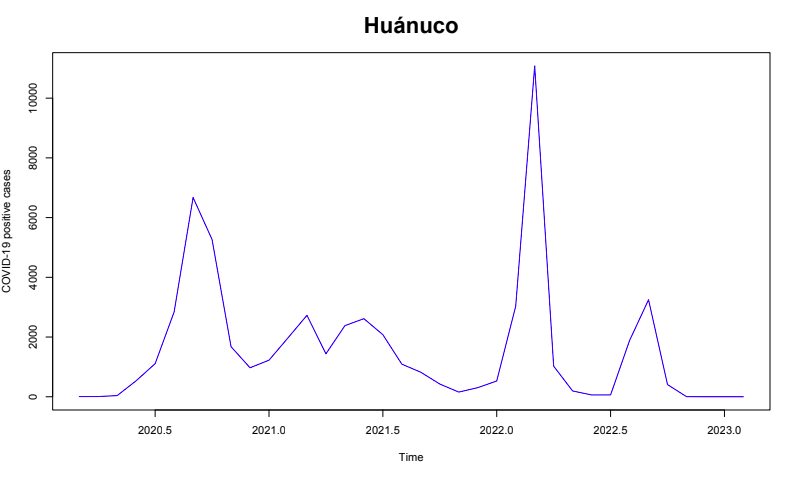 |
| 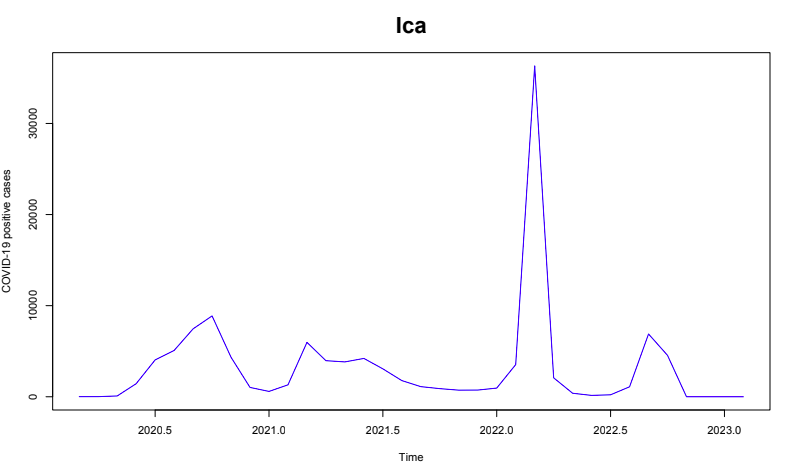 | 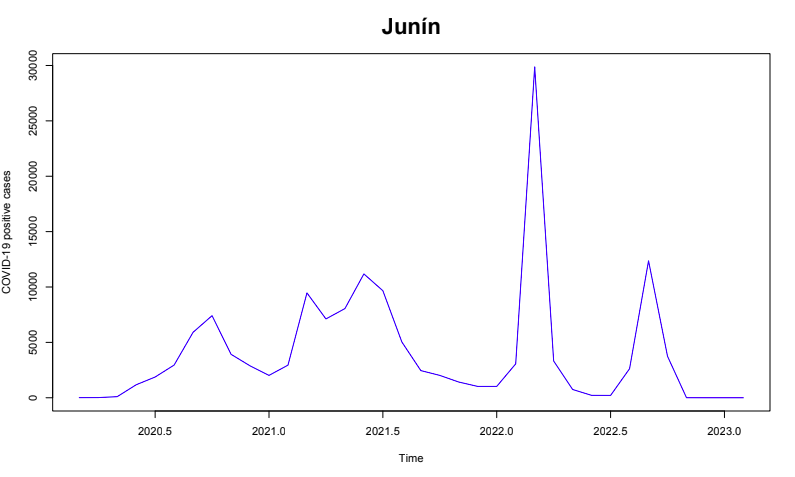 |
| 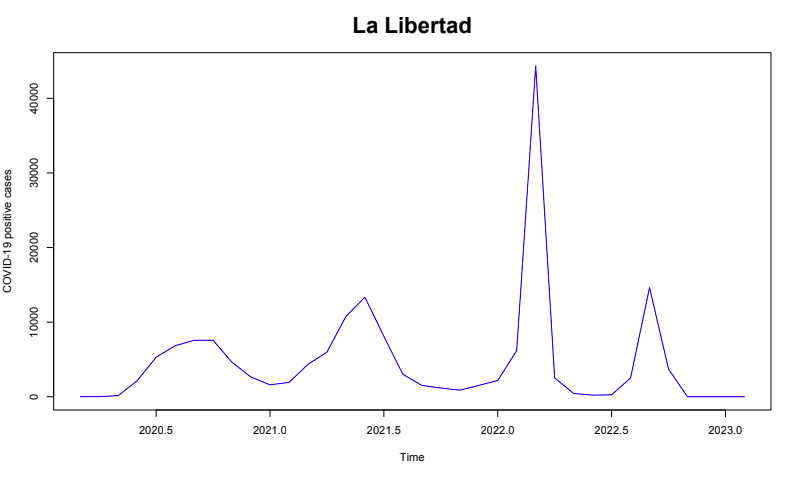 | 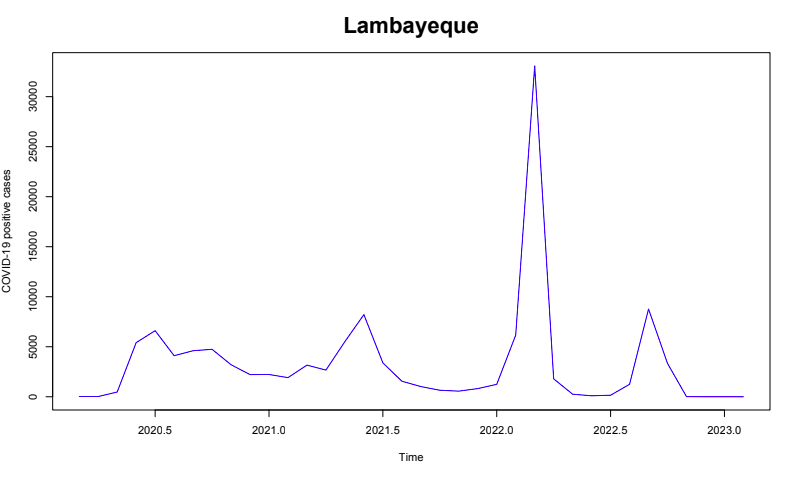 |
| 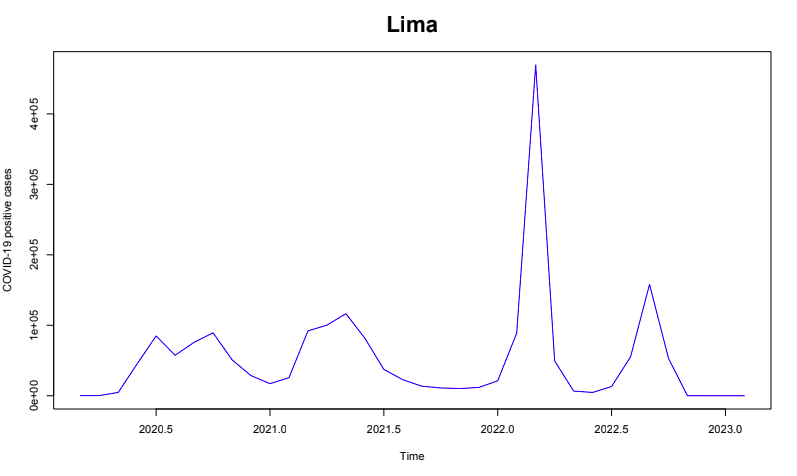 | 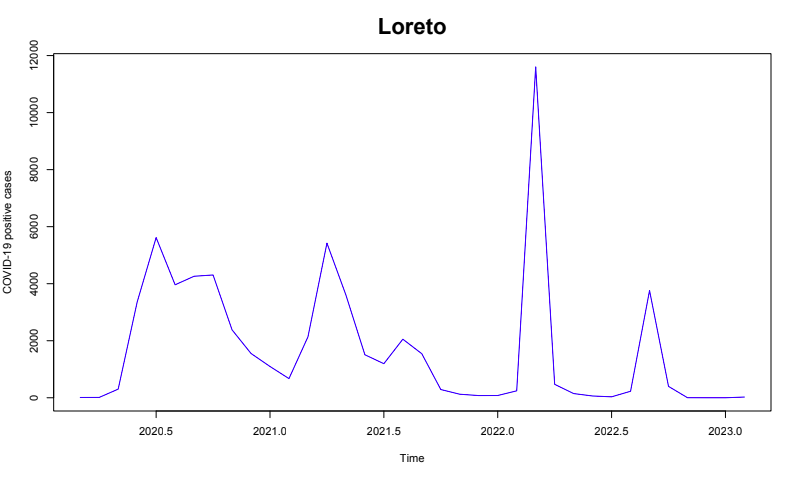 |
| 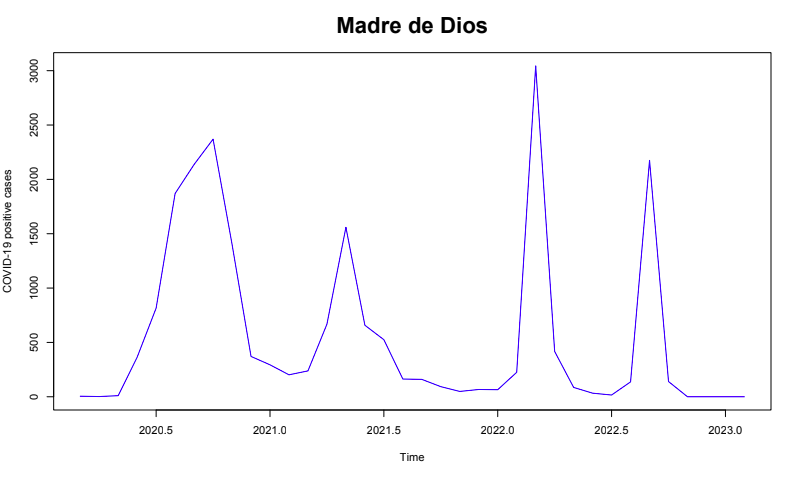 | 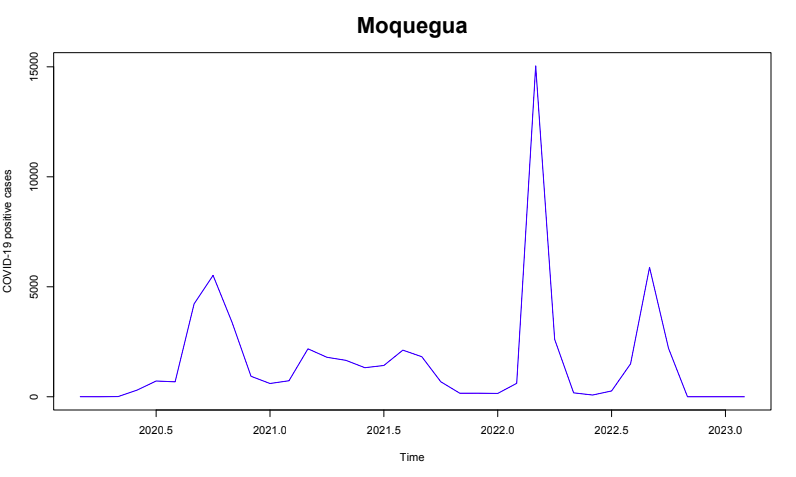 |
| 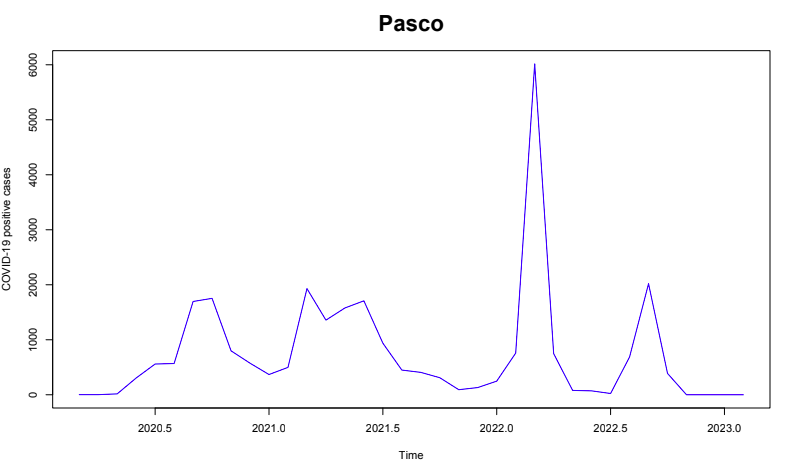 | 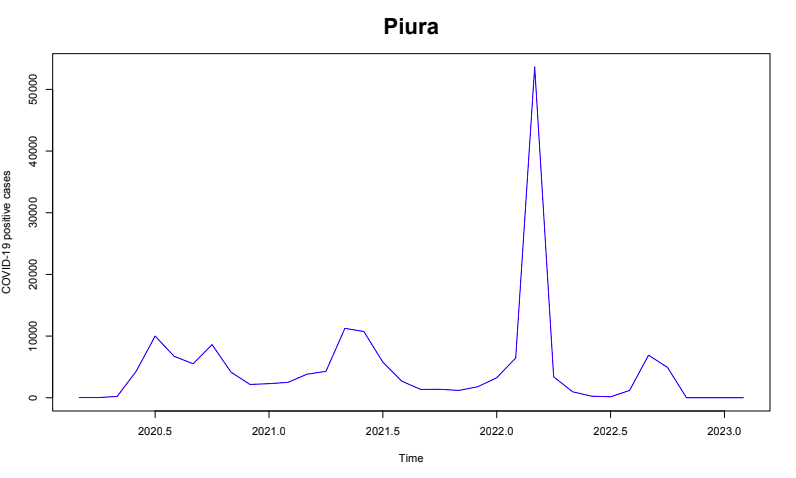 |
| 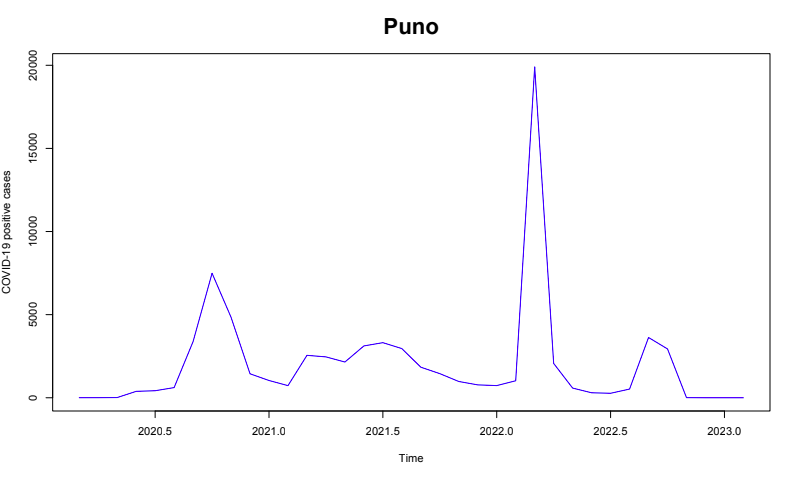 | 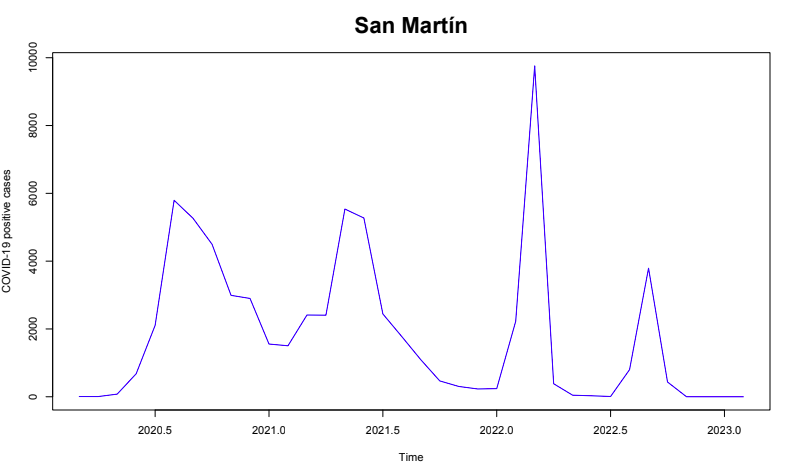 |
| 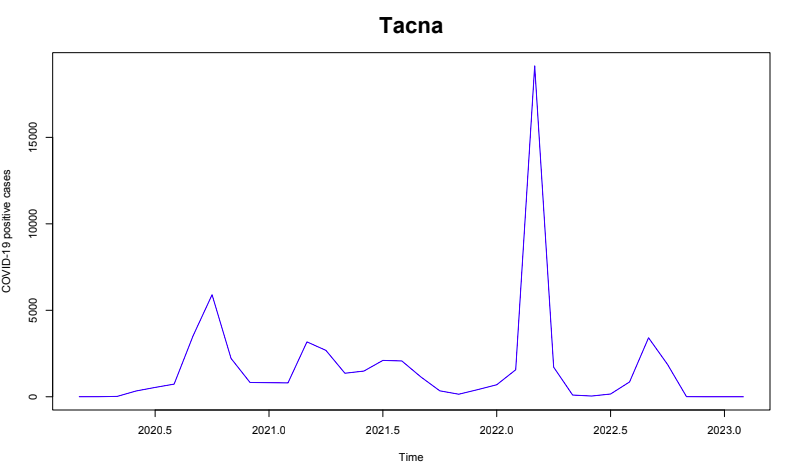 | 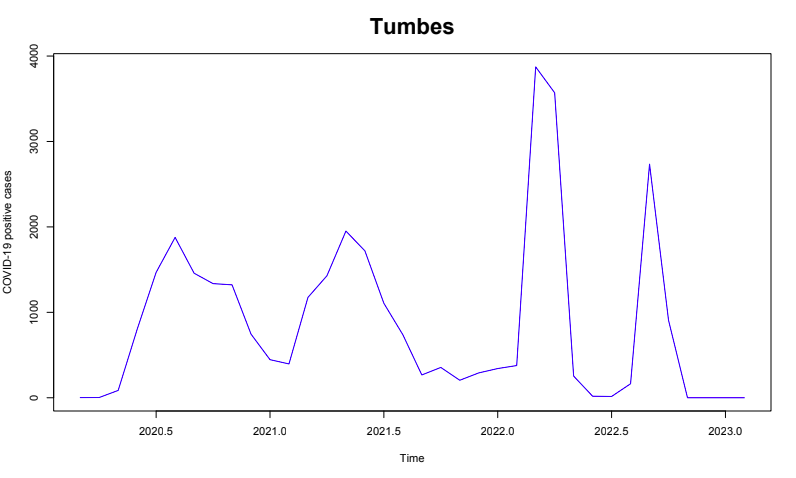 |
| 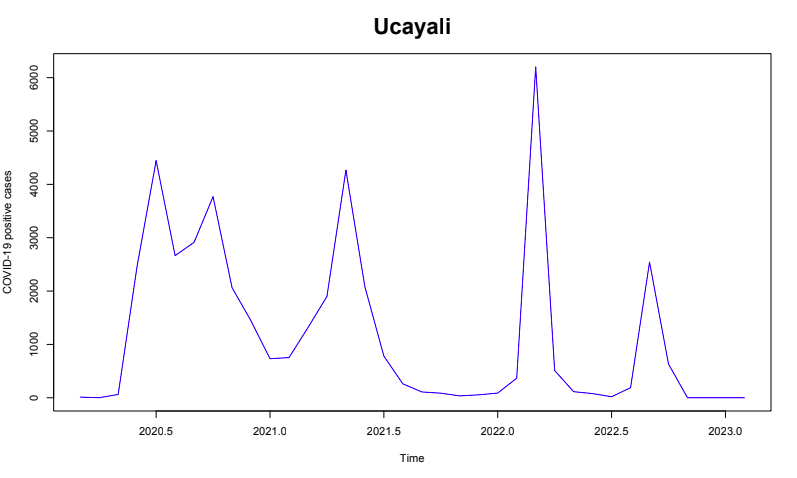 |  |

1. **DEATHS DUE TO COVID-19 BY DEPARTMENT DURING THE PERIOD 2020-2022.**

| 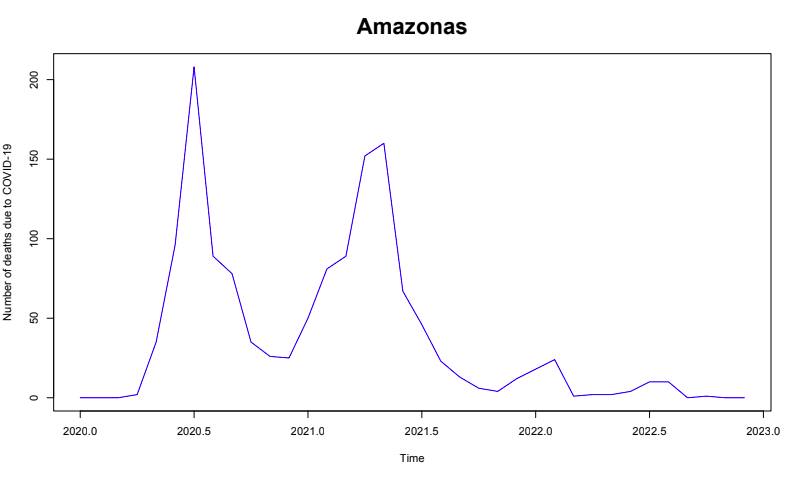 | 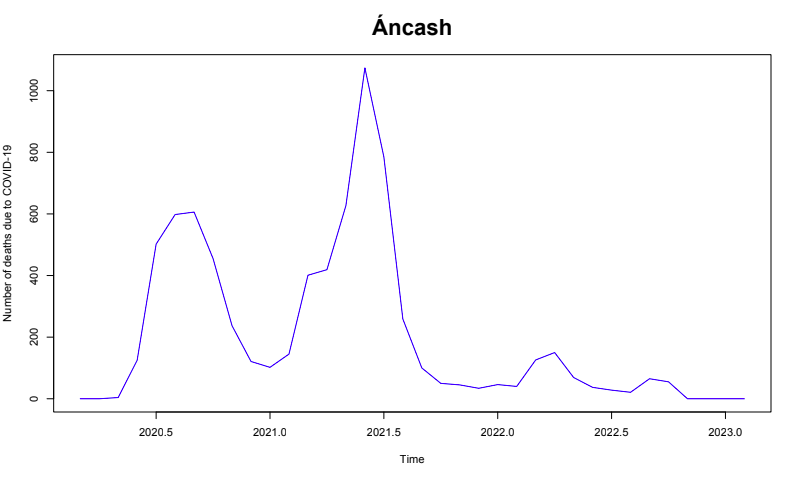 |
| --- | --- |
| 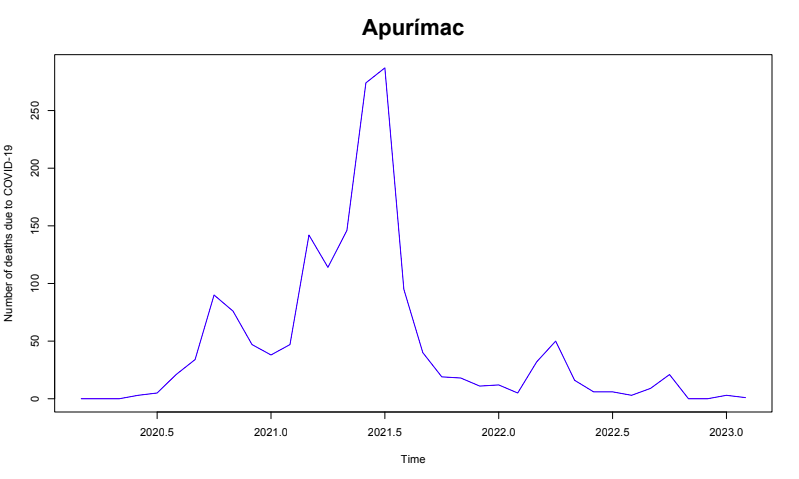 | 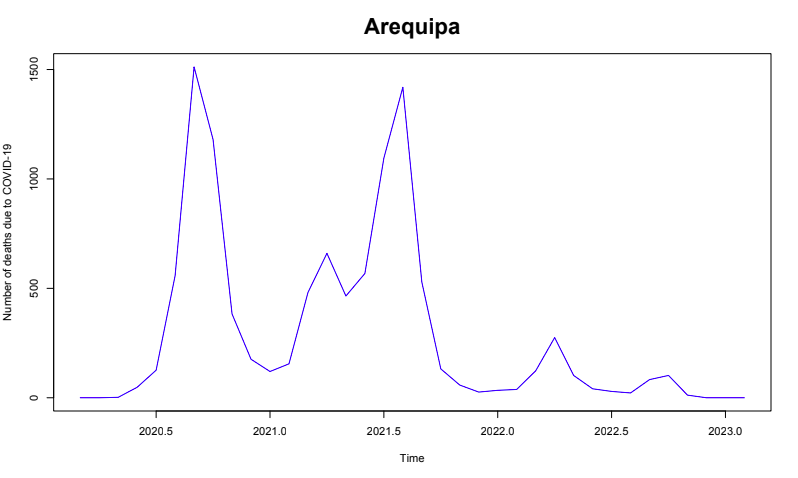 |
| 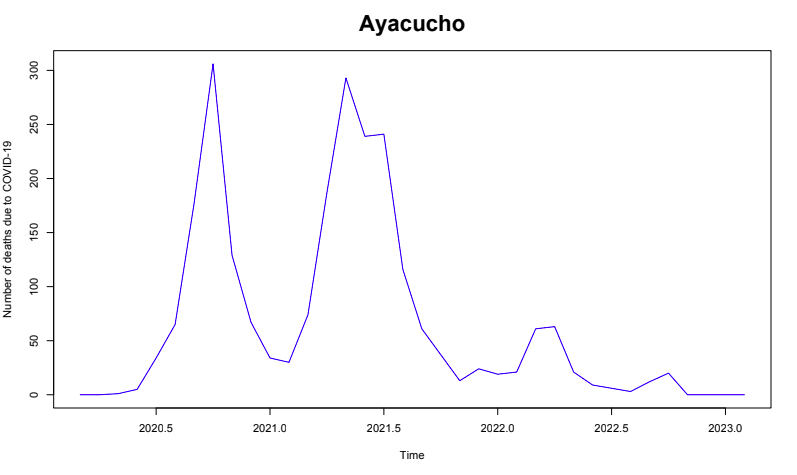 | 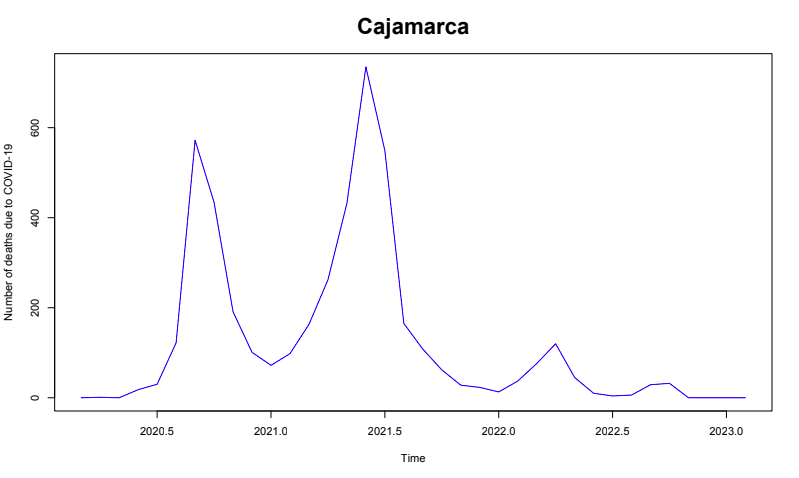 |
| 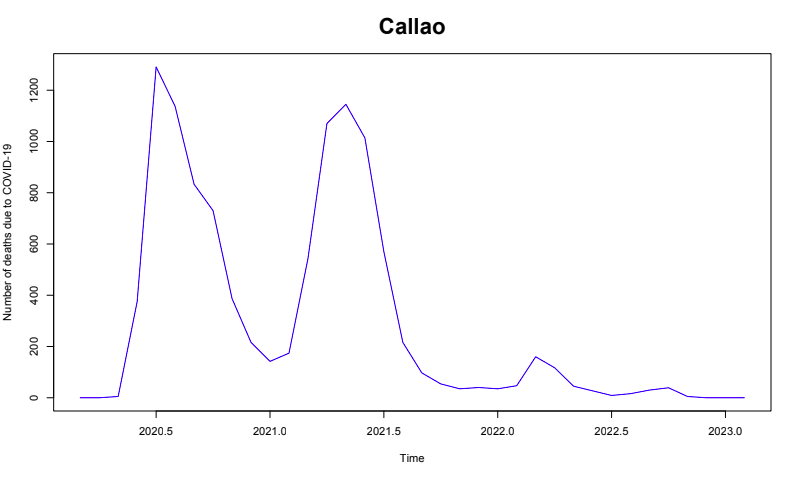 | 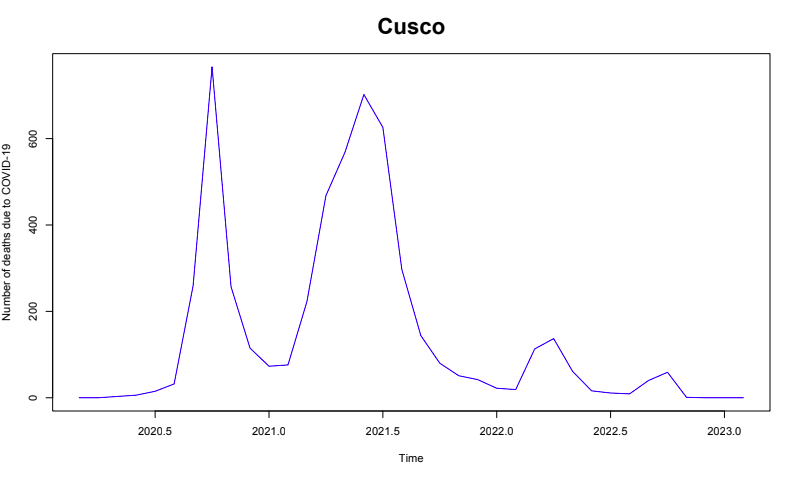 |
| 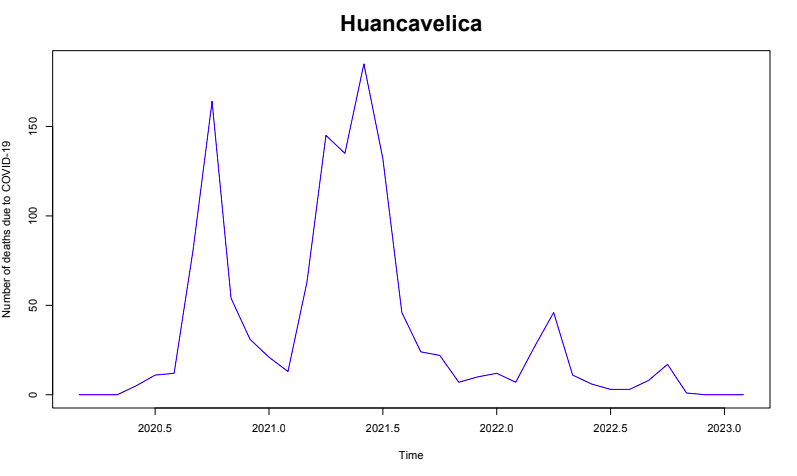 | 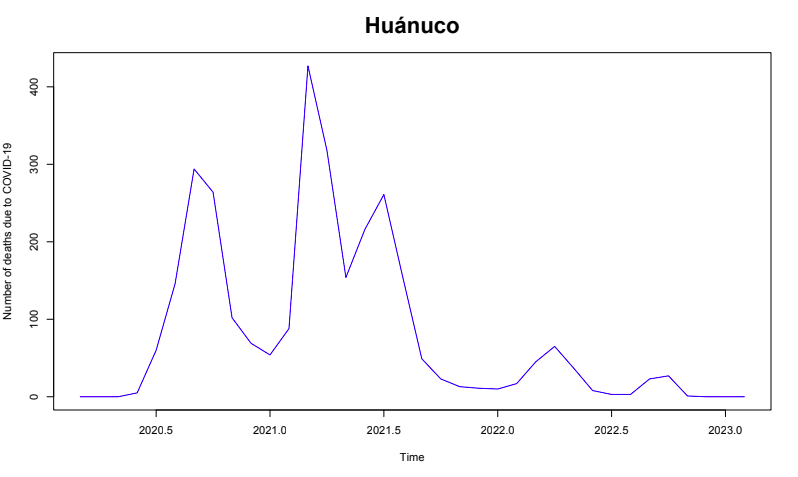 |
| 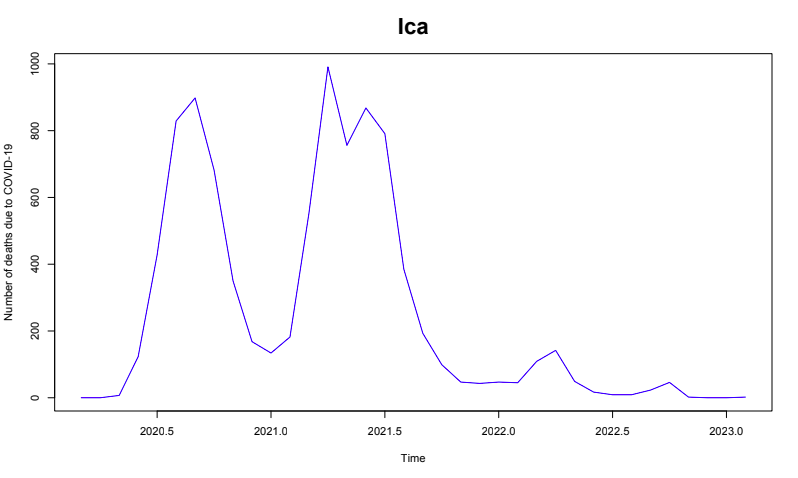 | 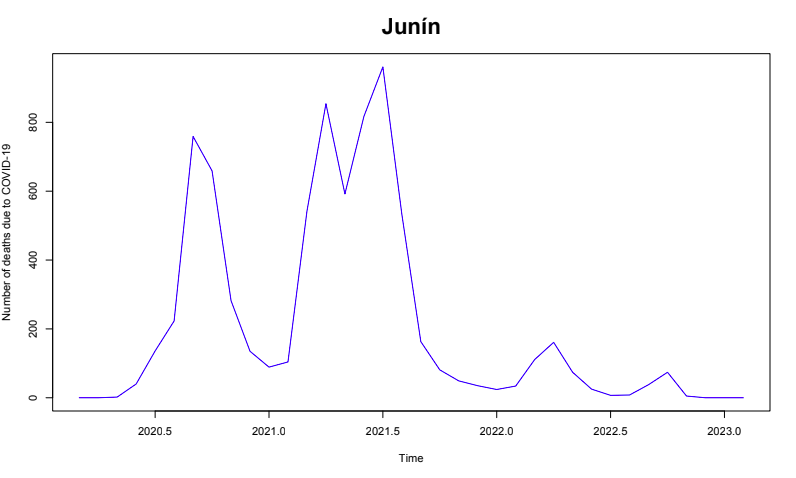 |
| 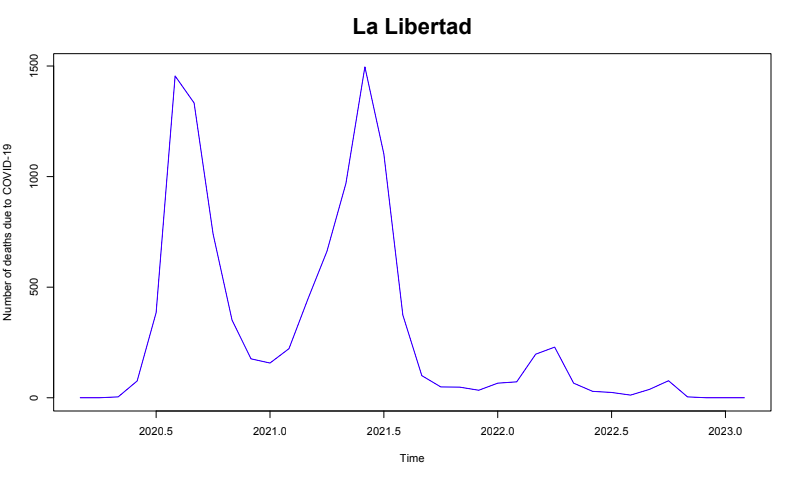 | 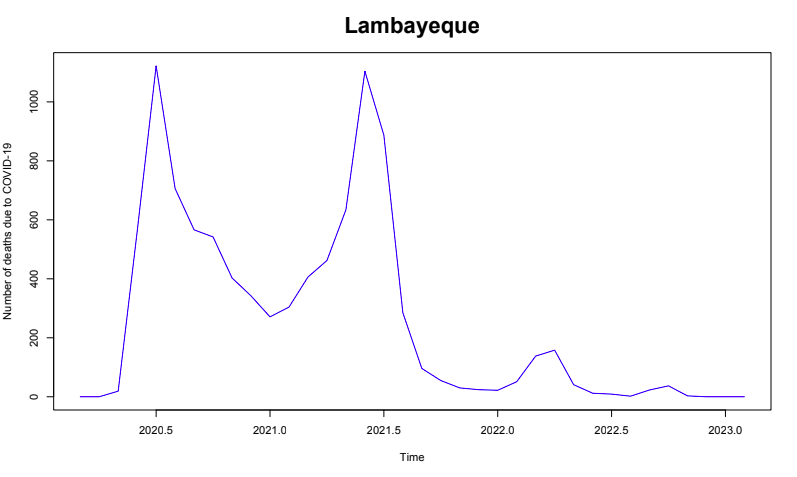 |
| 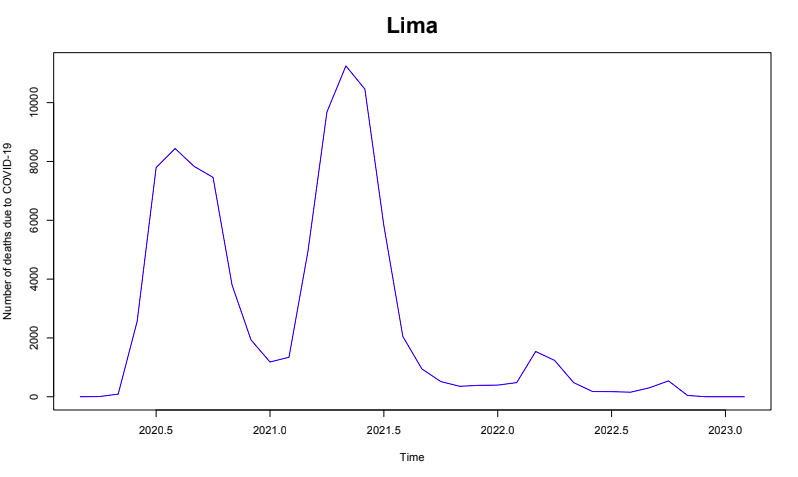 | 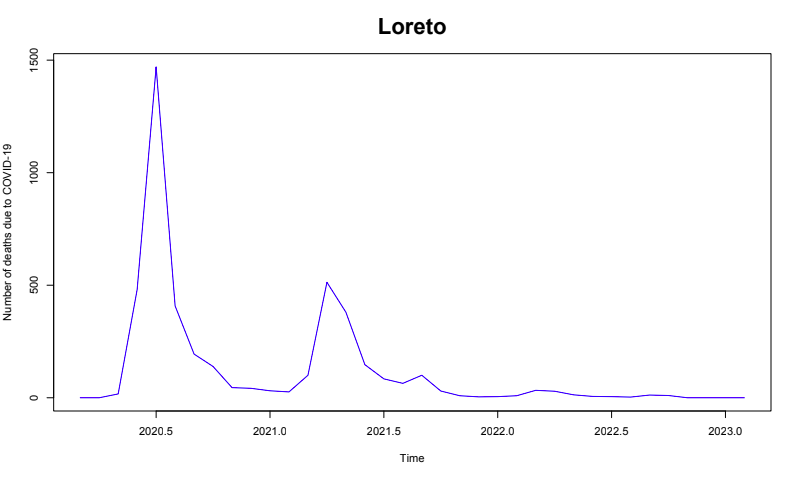 |
| 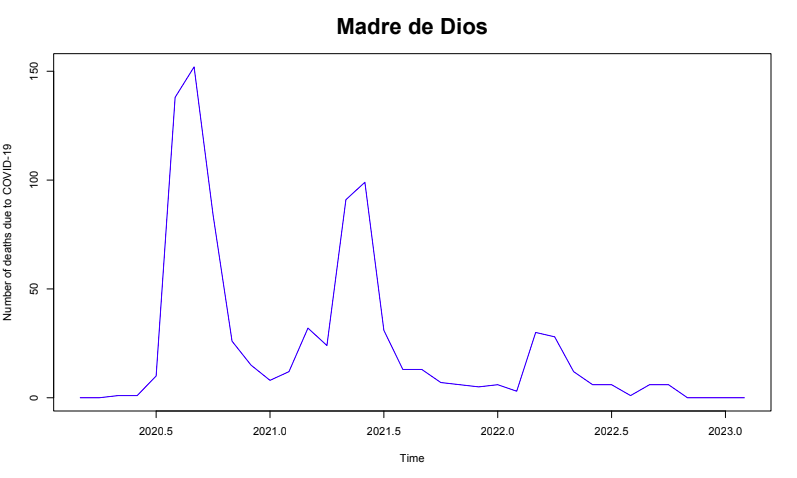 | 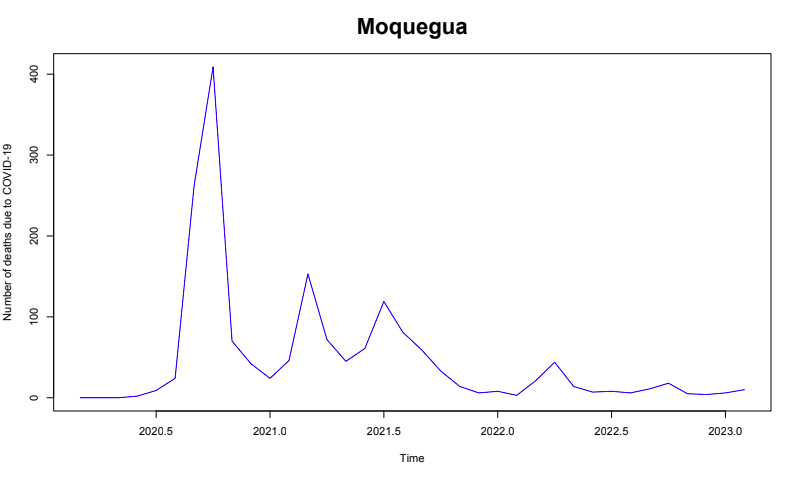 |
| 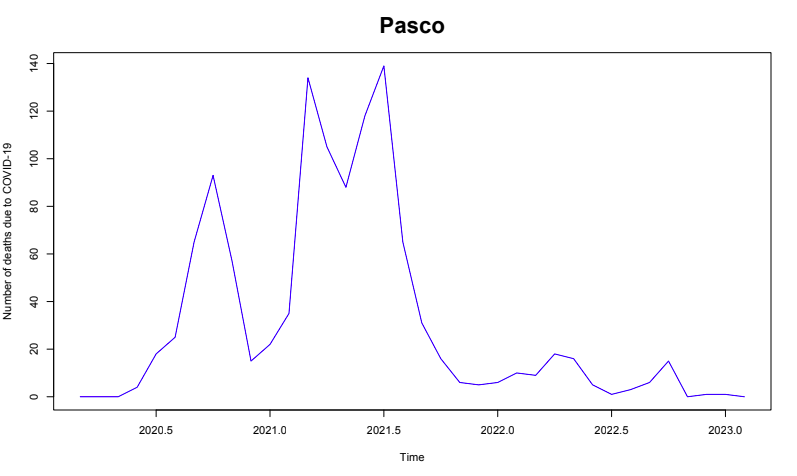 | 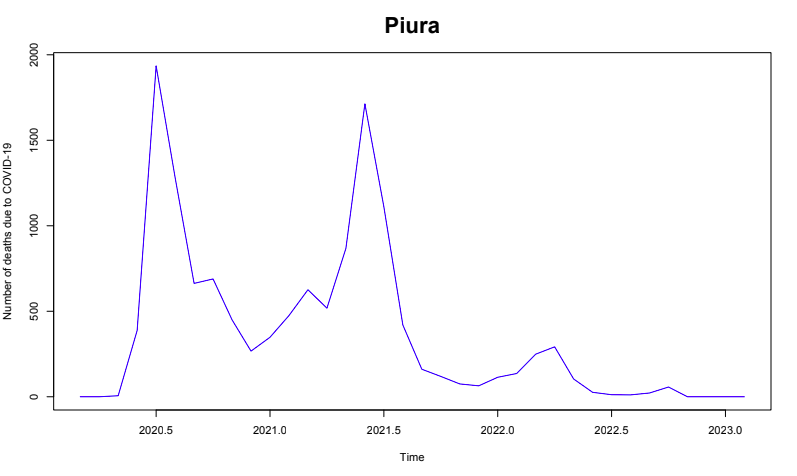 |
| 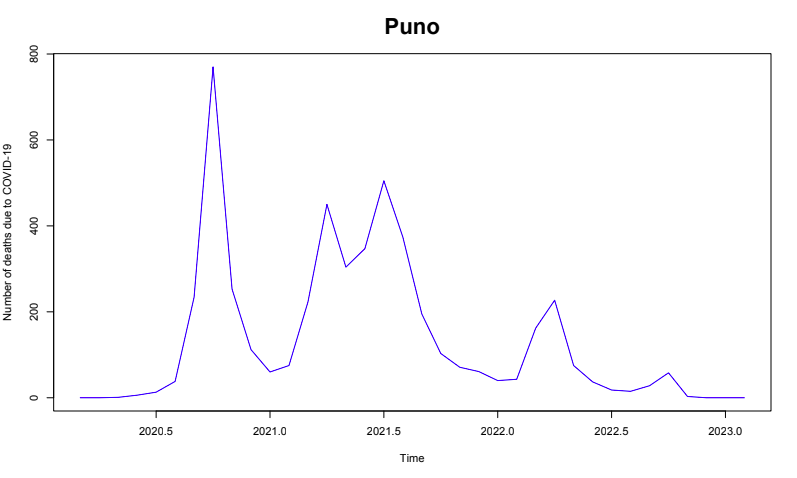 | 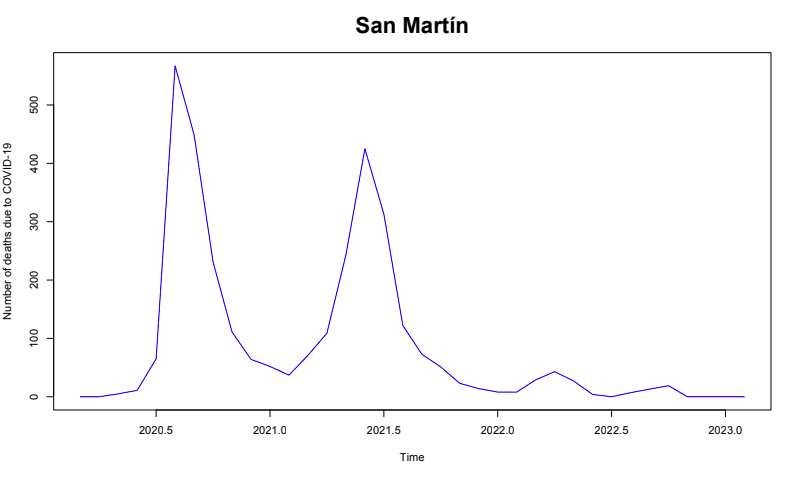 |
| 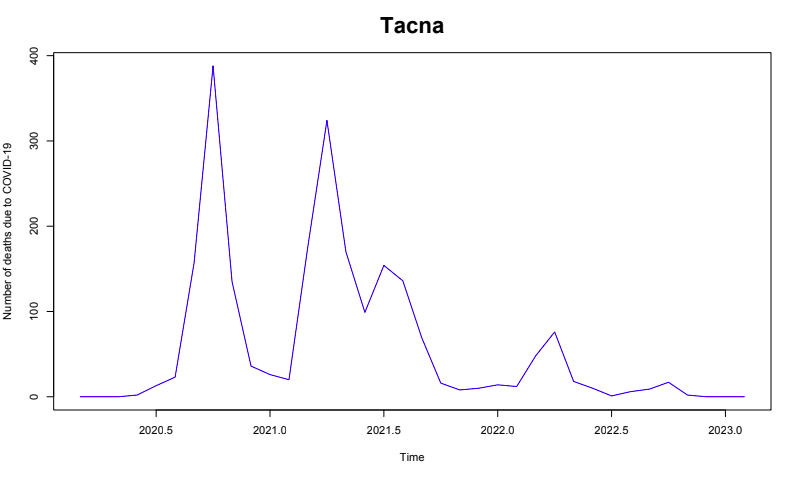 | 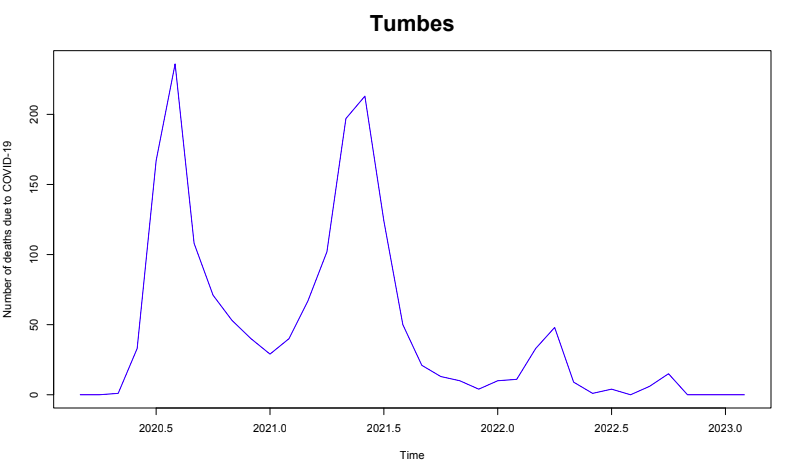 |
| 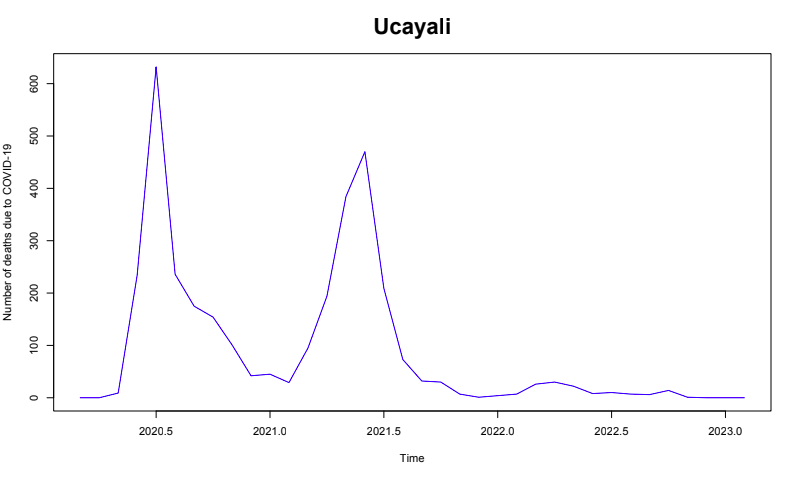 |  |

1. **COVID-19 POSITIVE CASES ACCORDING TO ALTITUDE BY DEPARTMENT DURING THE PERIOD 2020-2022.**

| AMAZONAS | ANCASH |
| --- | --- |
| 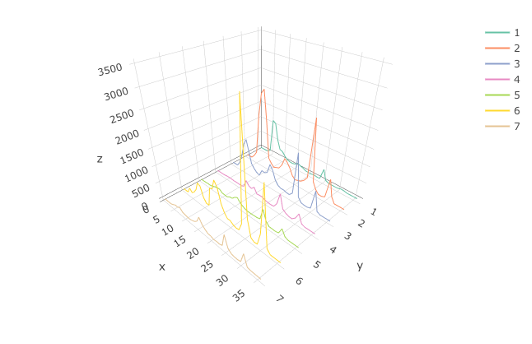 | 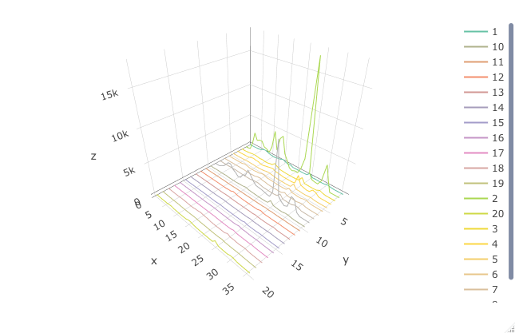 |
| APURIMAC | AREQUIPA |
| 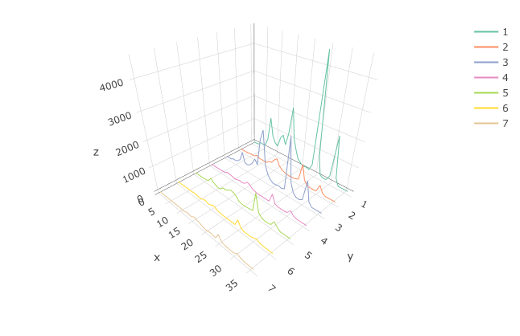 | 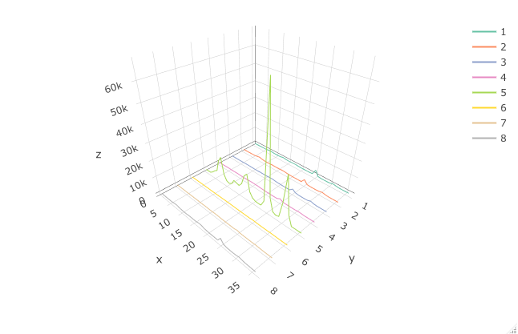 |
| AYACUCHO | CAJAMARCA |
| 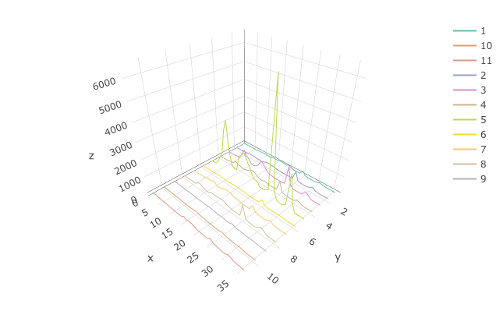 | 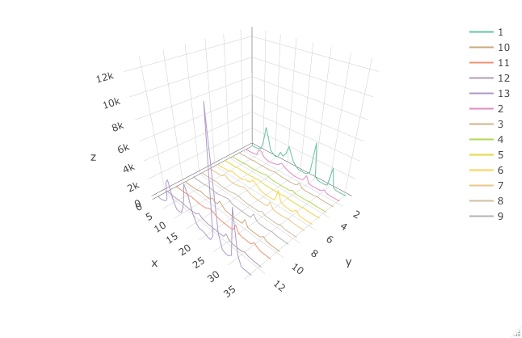 |
| CALLAO | CUSCO |
| 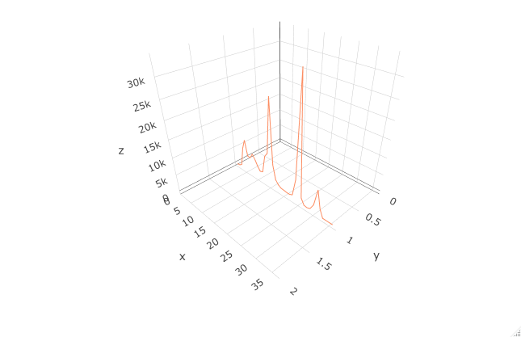 | 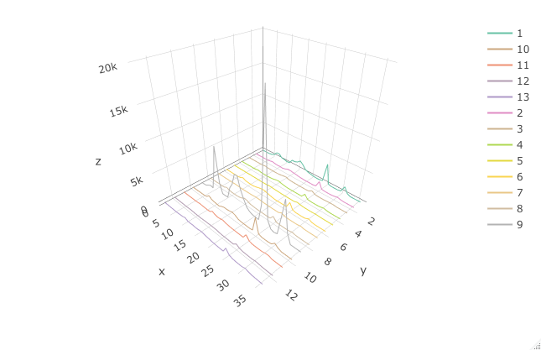 |
| HUANCAVELICA | HUÁNUCO |
| 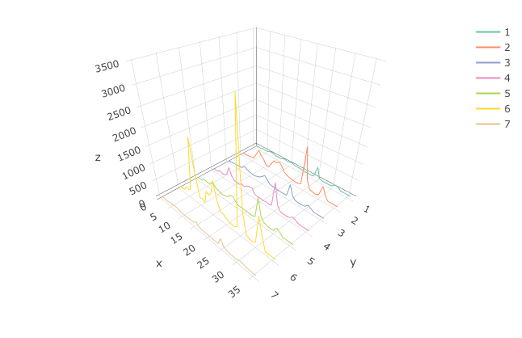 | 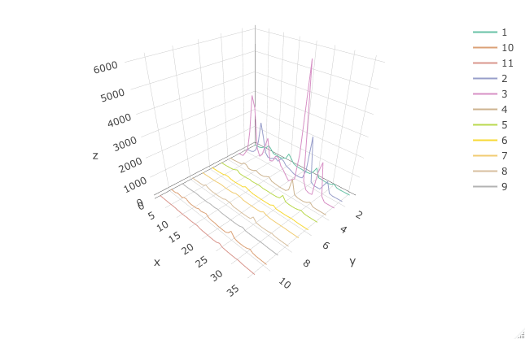 |
| ICA | JUNÍN |
| 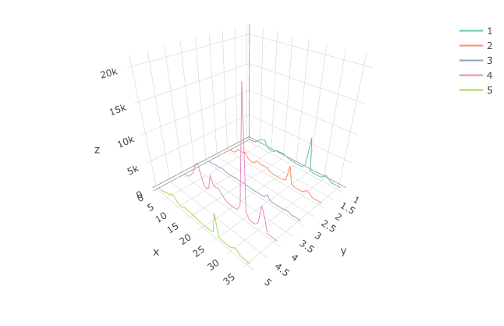 | 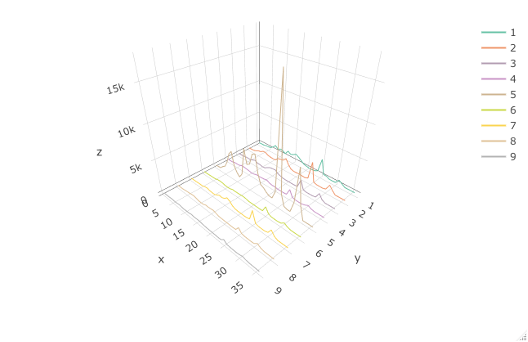 |
| LA LIBERTAD | LAMBAYEQUE |
| 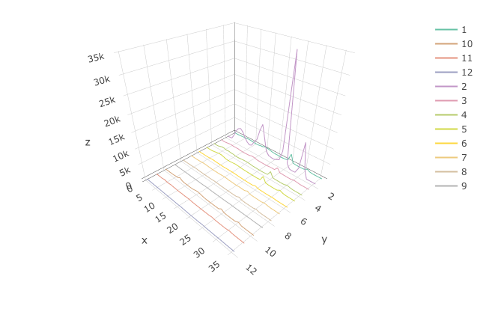 | 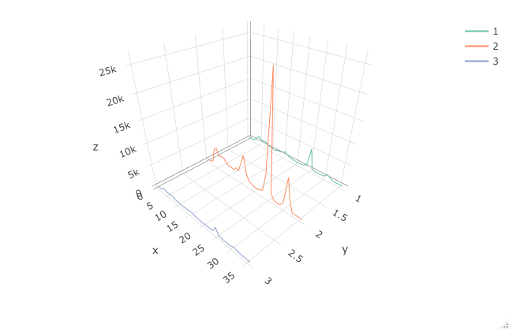 |
| LIMA | LORETO |
| 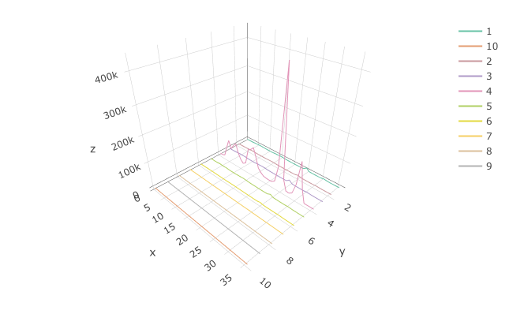 | 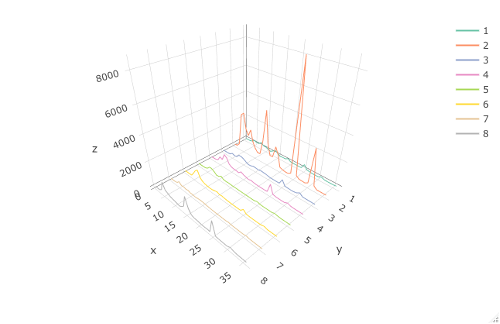 |
| MADRE DE DIOS | MOQUEGUA |
| 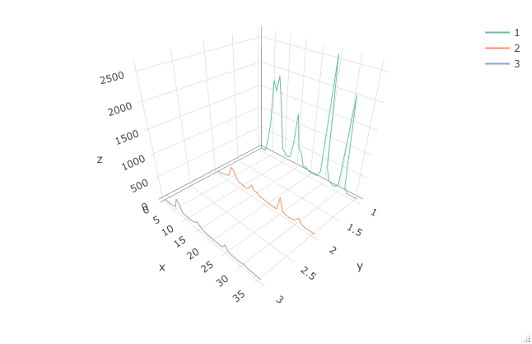 | 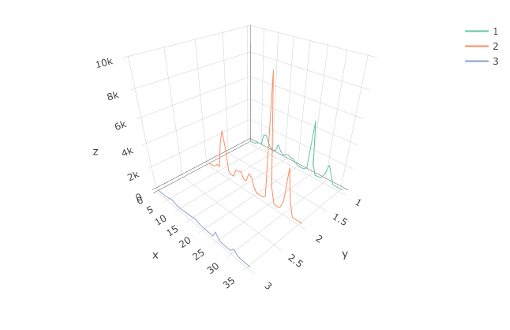 |
| PASCO | PIURA |
| 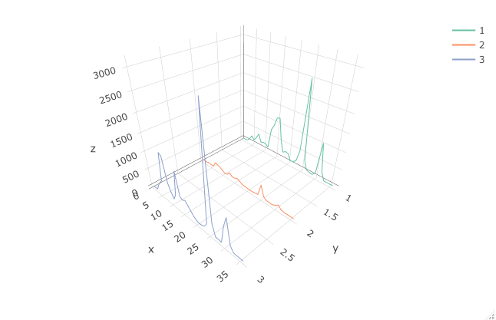 | 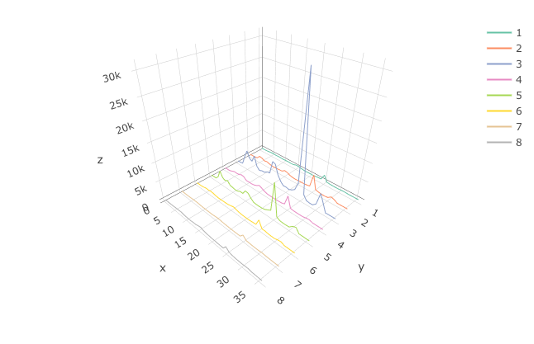 |
| PUNO | SAN MARTIN |
| 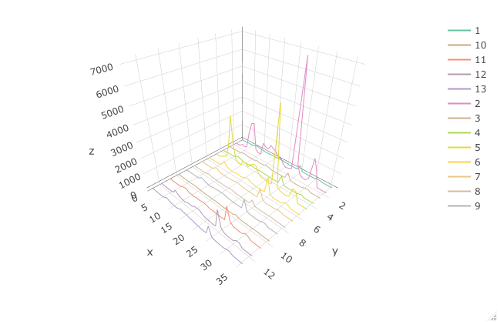 | 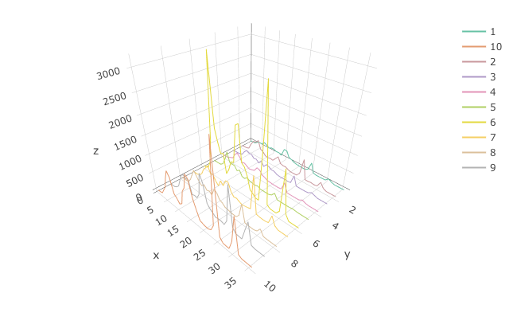 |
| TACNA | TUMBES |
| 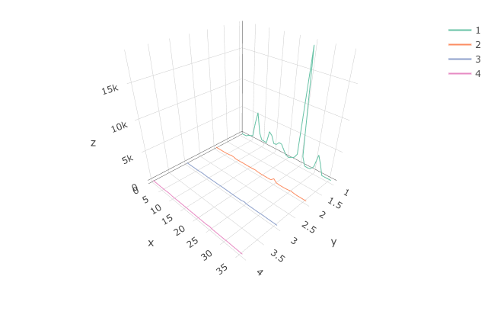 | 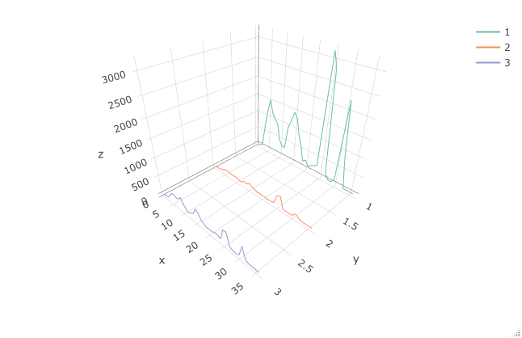 |
| UCAYALI | |
| 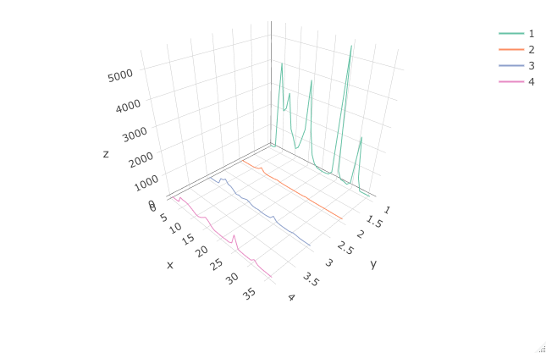 | |

1. **COVID-19 POSITIVE CASES ACCORDING TO POPULATION DENSITY BY DEPARTMENT DURING THE PERIOD 2020-2022.**

| AMAZONAS | ANCASH |
| --- | --- |
| 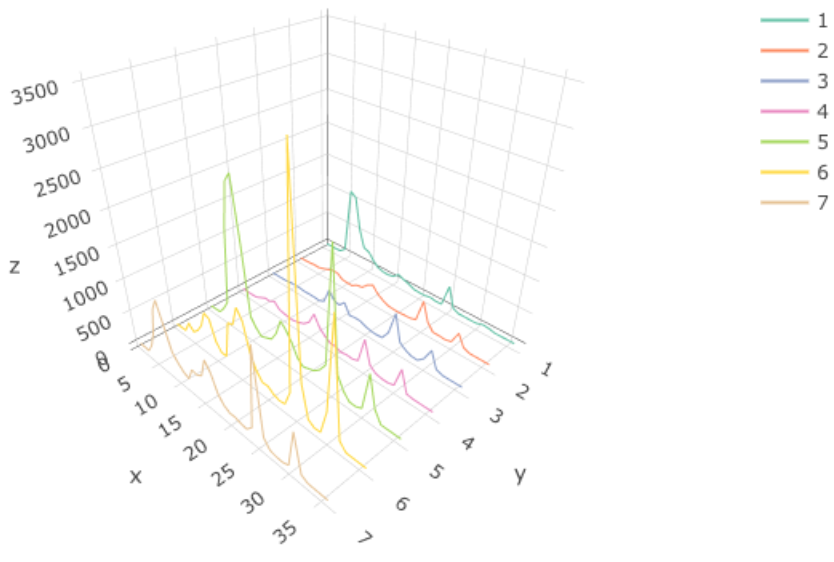 | 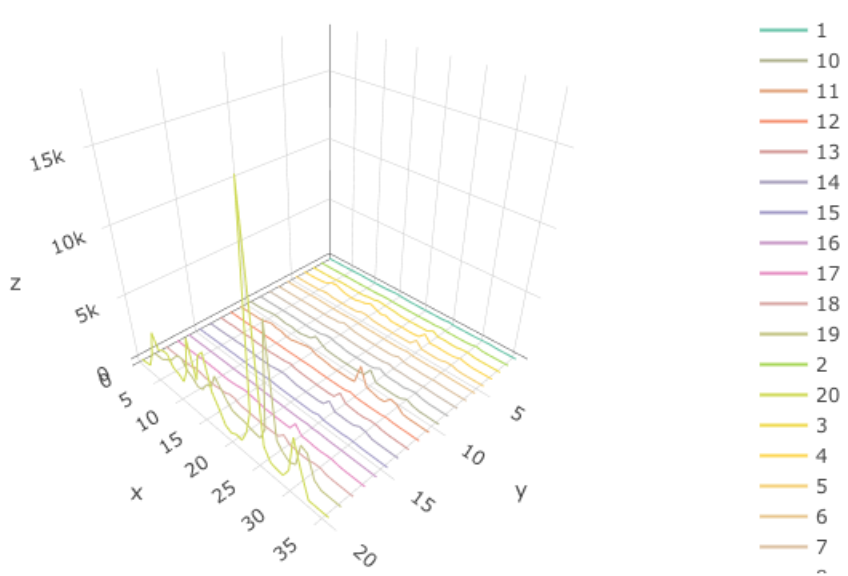 |
| APURIMAC | AREQUIPA |
| 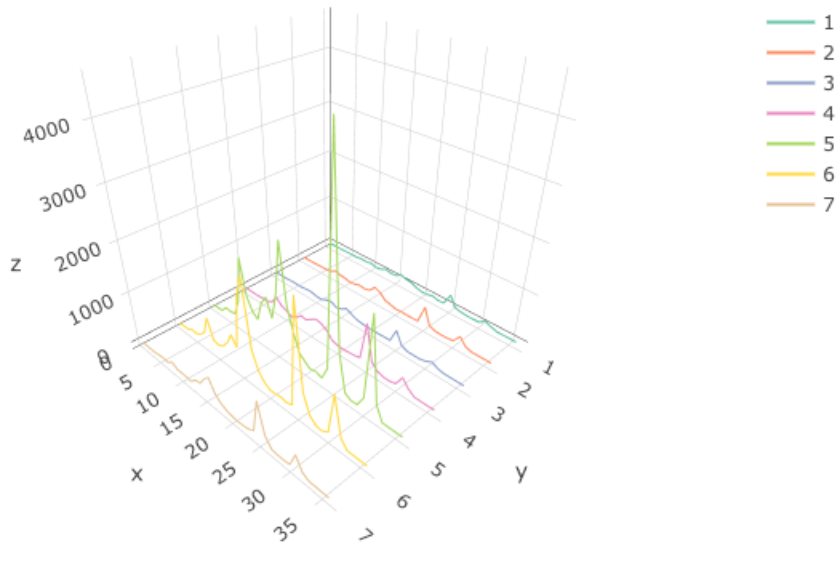 | 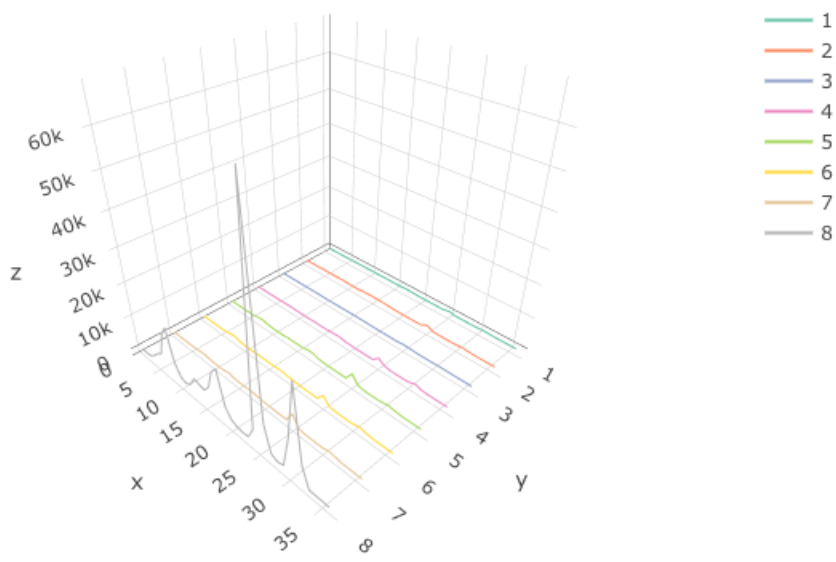 |
| AYACUCHO | CAJAMARCA |
| 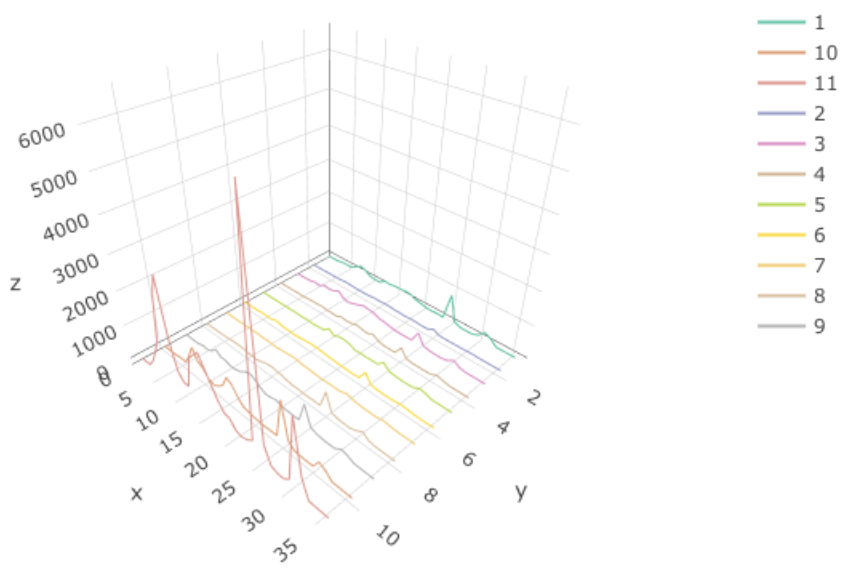 | 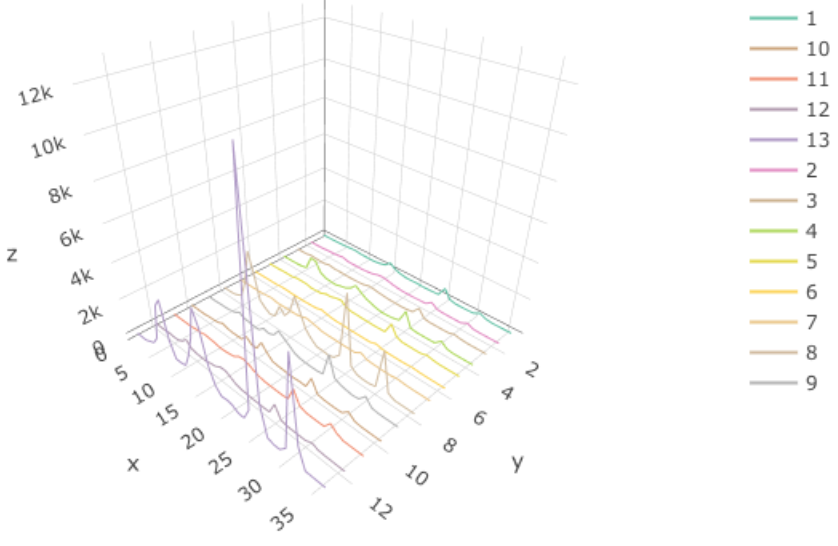 |
| CALLAO | CUSCO |
| 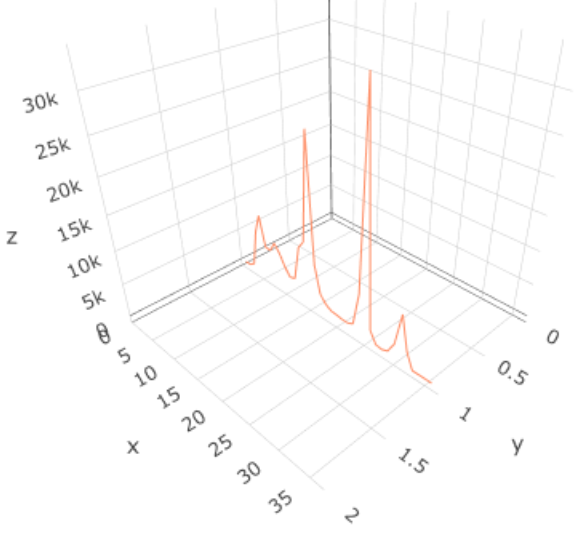 | 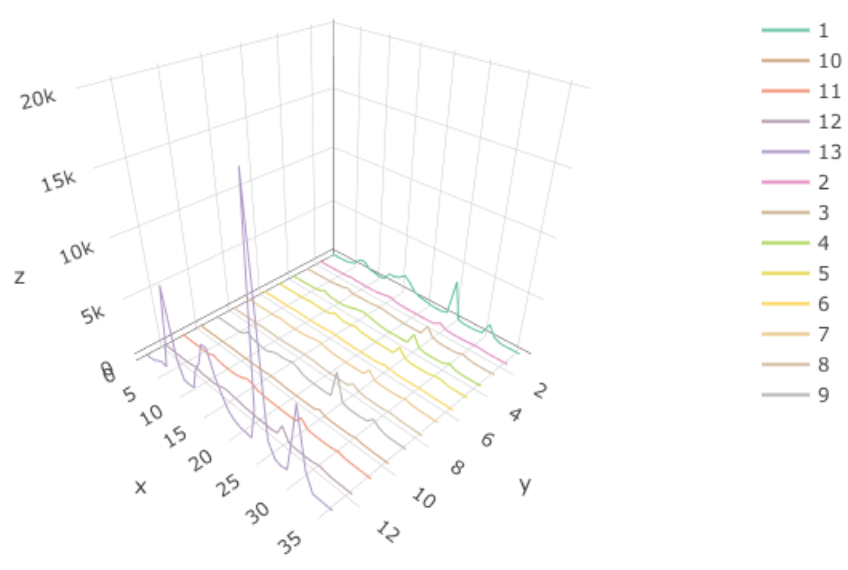 |
| HUANCAVELICA | HUÁNUCO |
| 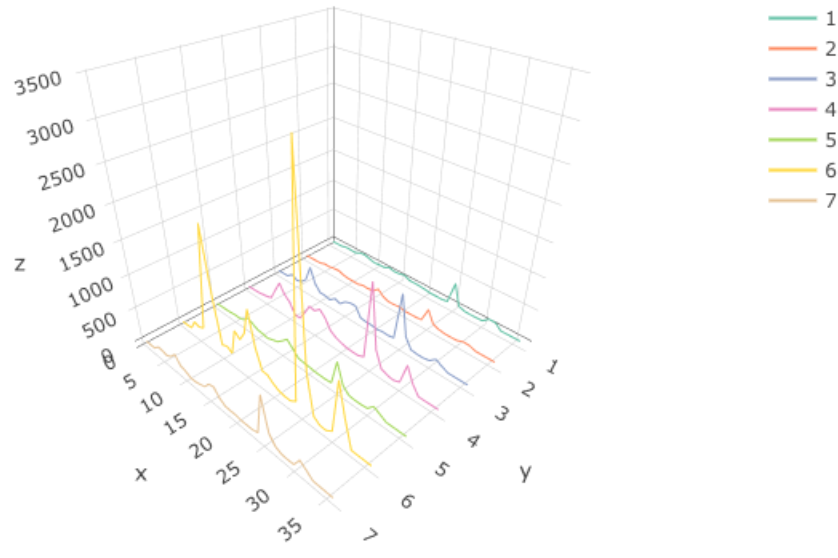 | 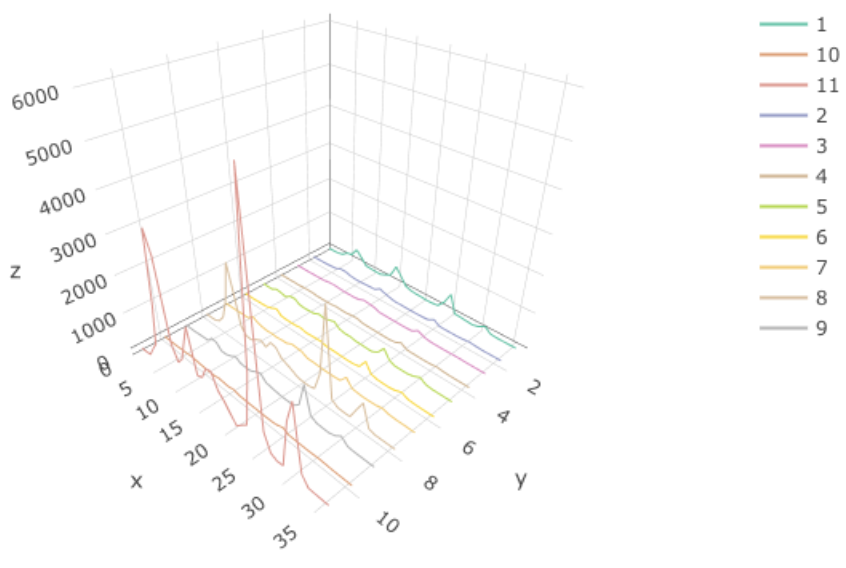 |
| ICA | JUNÍN |
| 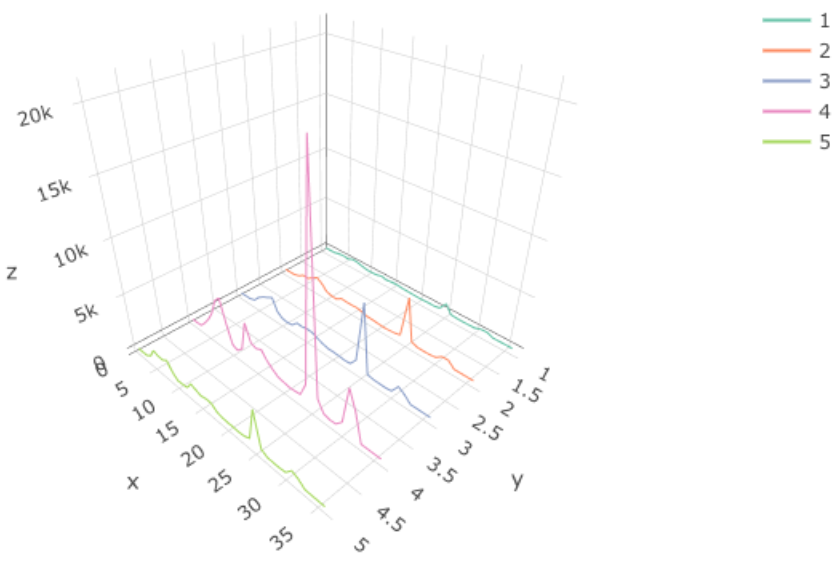 | 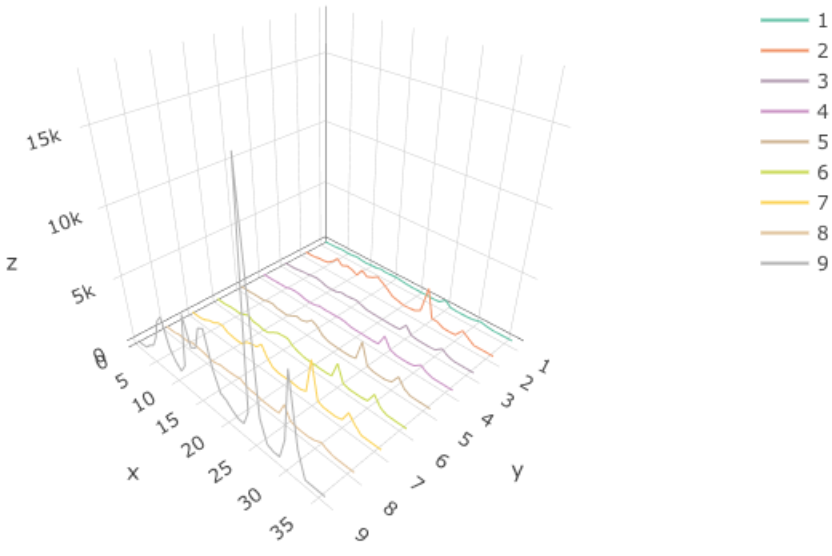 |
| LA LIBERTAD | LAMBAYEQUE |
| 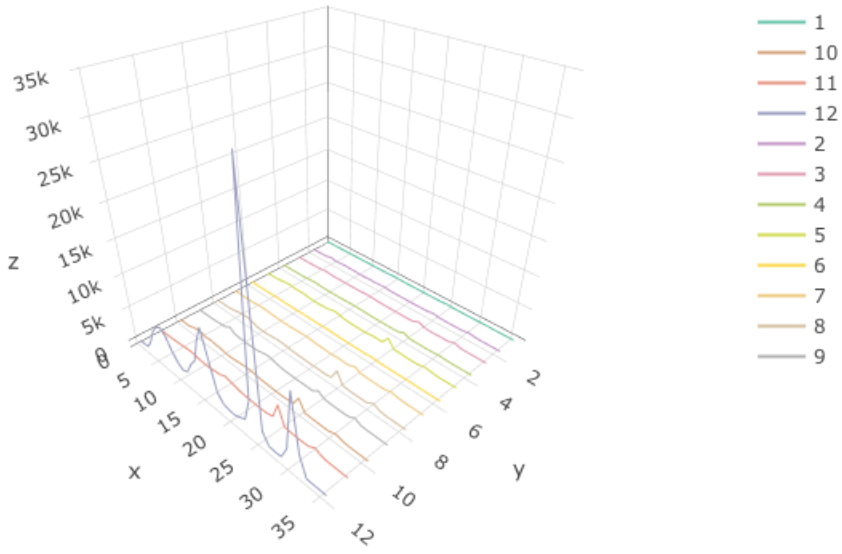 | 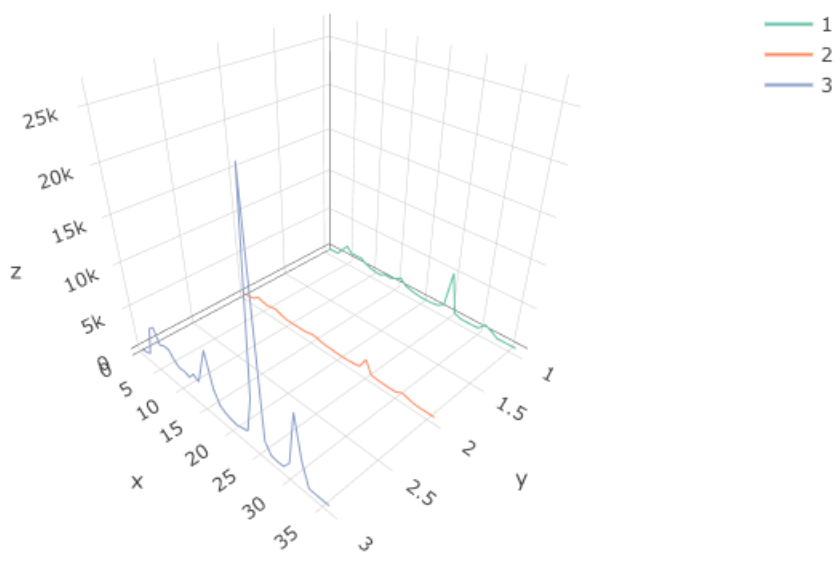 |
| LIMA | LORETO |
| 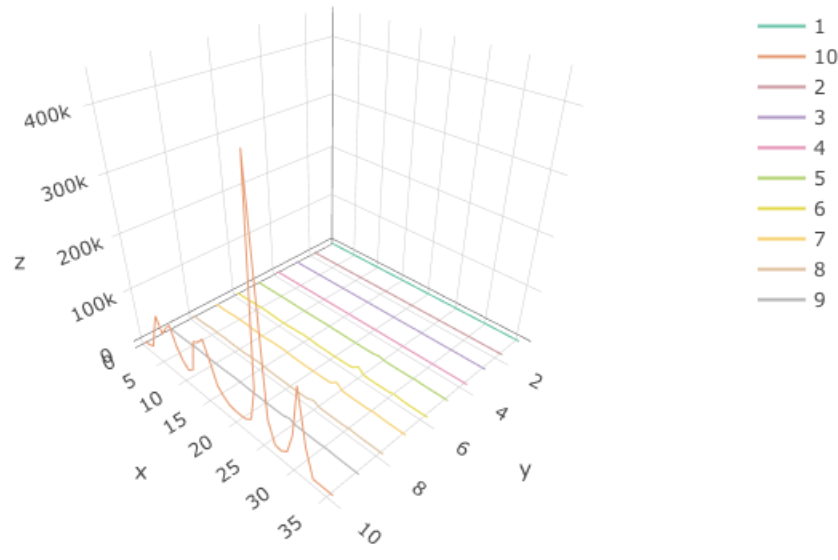 | 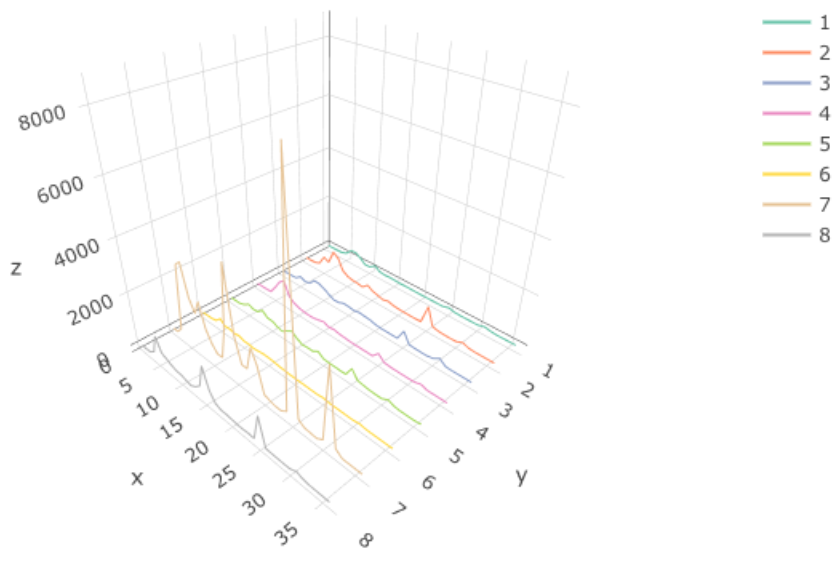 |
| MADRE DE DIOS | MOQUEGUA |
| 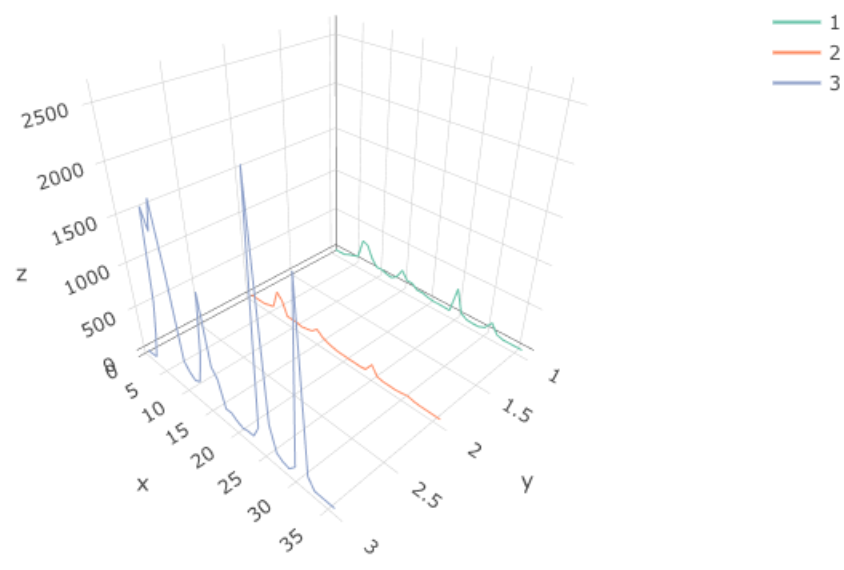 | 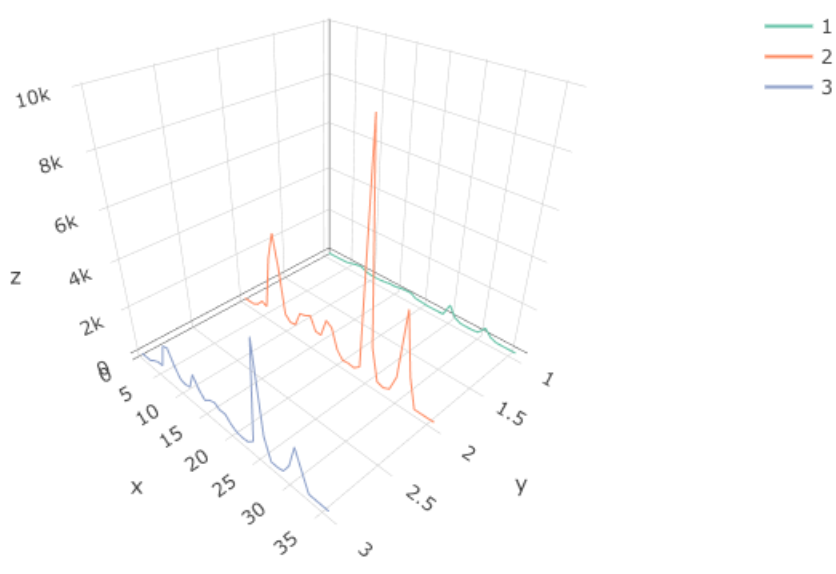 |
| PASCO | PIURA |
| 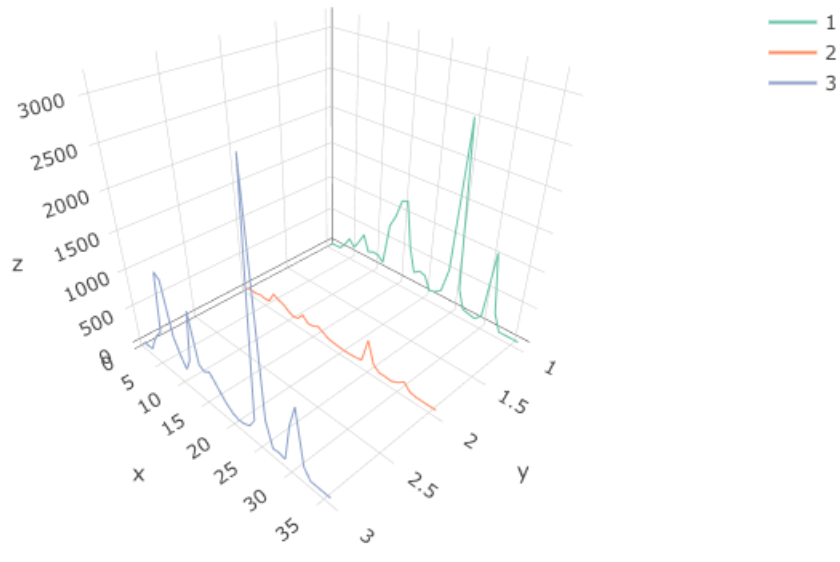 | 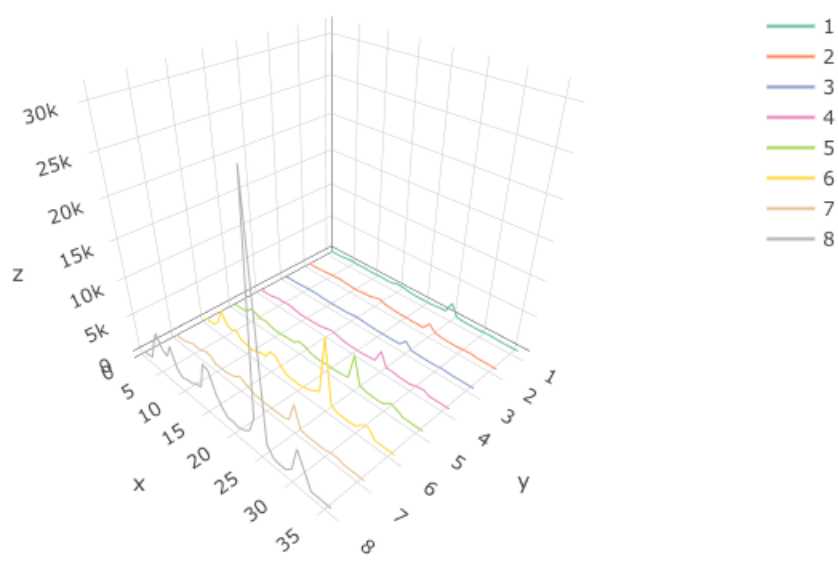 |
| PUNO | SAN MARTIN |
| 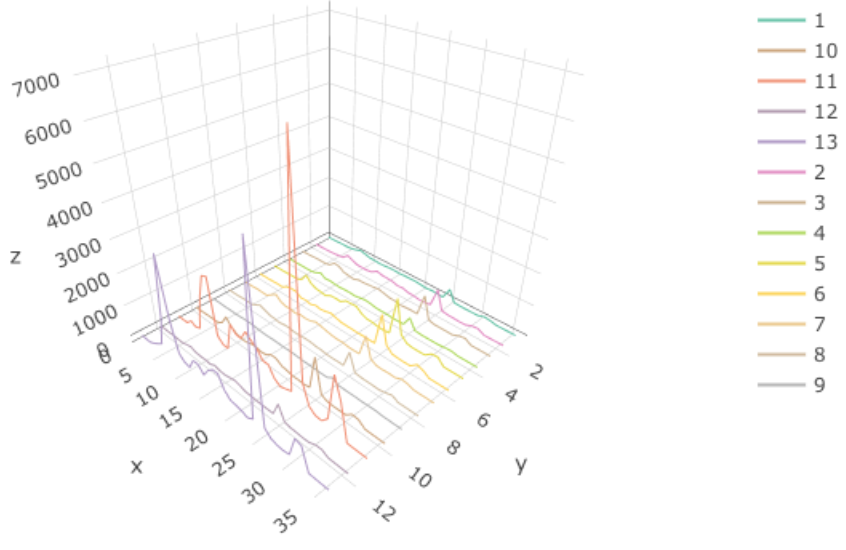 | 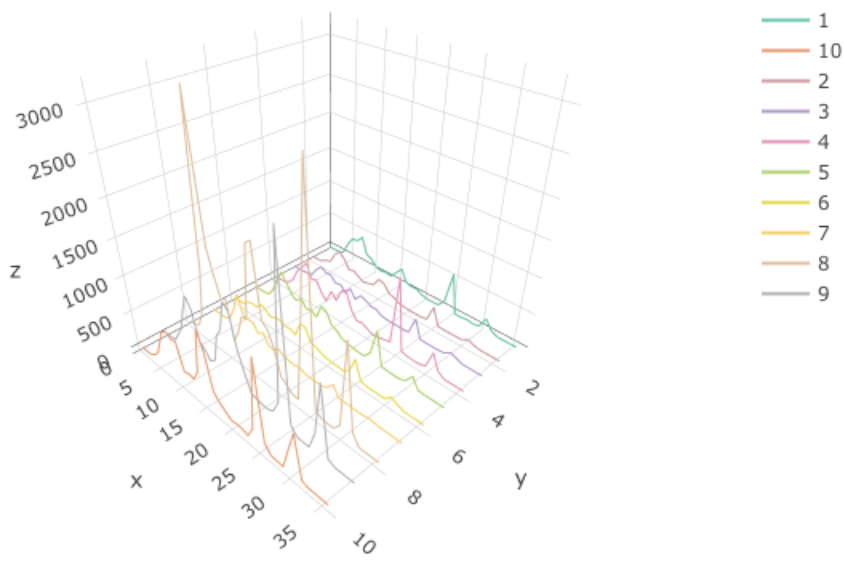 |
| TACNA | TUMBES |
| 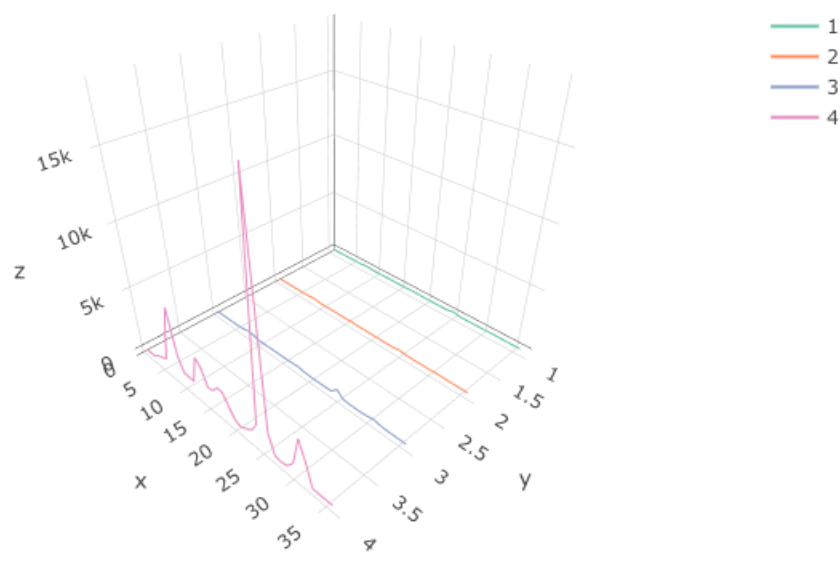 | 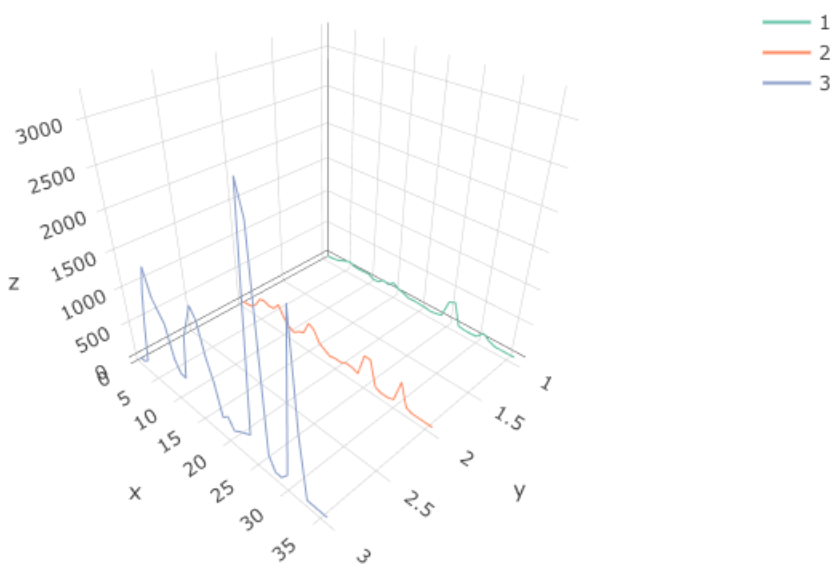 |
| UCAYALI | |
| 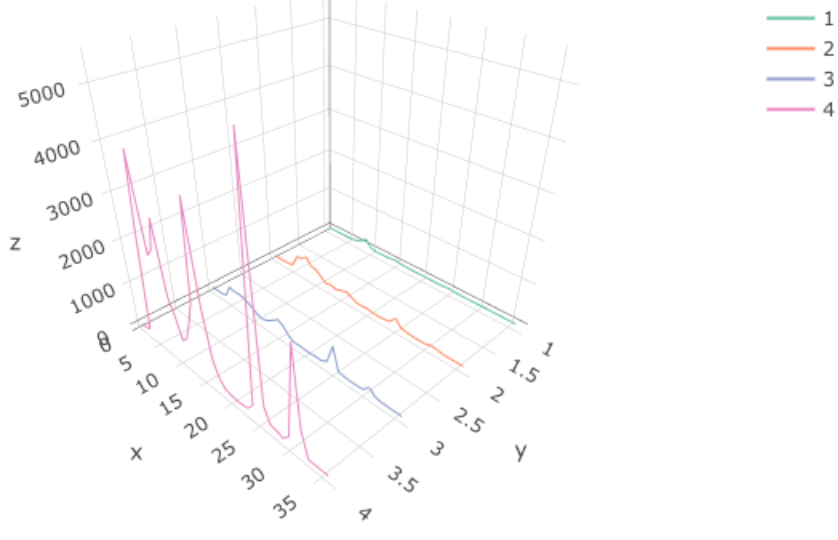 | |

1. **COVID-19 POSITIVE CASES ACCORDING TO PERCENTAGE OF POPULATION IN TOTAL POVERTY BY DEPARTMENT DURING THE PERIOD 2020-2022.**

| AMAZONAS | ANCASH |
| --- | --- |
|  |  |
| APURIMAC | AREQUIPA |
|  |  |
| AYACUCHO | CAJAMARCA |
|  |  |
| CALLAO | CUSCO |
|  |  |
| HUANCAVELICA | HUÁNUCO |
|  |  |
| ICA | JUNÍN |
|  |  |
| LA LIBERTAD | LAMBAYEQUE |
|  |  |
| LIMA | LORETO |
|  |  |
| MADRE DE DIOS | MOQUEGUA |
|  |  |
| PASCO | PIURA |
|  |  |
| PUNO | SAN MARTIN |
|  |  |
| TACNA | TUMBES |
|  |  |
| UCAYALI | |
|  | |

1. **DEATHS DUE TO COVID-19 ACCORDING TO ALTITUDE BY DEPARTMENT DURING THE PERIOD 2020-2022.**

| AMAZONAS | ANCASH |
| --- | --- |
|  |  |
| APURIMAC | AREQUIPA |
|  |  |
| AYACUCHO | CAJAMARCA |
|  |  |
| CALLAO | CUSCO |
|  |  |
| HUANCAVELICA | HUÁNUCO |
|  |  |
| ICA | JUNÍN |
|  |  |
| LA LIBERTAD | LAMBAYEQUE |
|  |  |
| LIMA | LORETO |
|  |  |
| MADRE DE DIOS | MOQUEGUA |
|  |  |
| PASCO | PIURA |
|  |  |
| PUNO | SAN MARTIN |
|  |  |
| TACNA | TUMBES |
|  |  |
| UCAYALI | |
|  | |

1. **DEATHS DUE TO COVID-19 ACCORDING TO POPULATION DENSITY BY DEPARTMENT DURING THE PERIOD 2020-2022.**

| AMAZONAS | ANCASH |
| --- | --- |
|  |  |
| APURIMAC | AREQUIPA |
|  |  |
| AYACUCHO | CAJAMARCA |
|  |  |
| CALLAO | CUSCO |
|  |  |
| HUANCAVELICA | HUÁNUCO |
|  |  |
| ICA | JUNÍN |
|  |  |
| LA LIBERTAD | LAMBAYEQUE |
|  |  |
| LIMA | LORETO |
|  |  |
| MADRE DE DIOS | MOQUEGUA |
|  |  |
| PASCO | PIURA |
|  |  |
| PUNO | SAN MARTIN |
|  |  |
| TACNA | TUMBES |
|  |  |
| UCAYALI | |
|  | |

1. **DEATHS DUE TO COVID-19 ACCORDING TO PERCENTAGE OF POPULATION IN TOTAL POVERTY BY DEPARTMENT DURING THE PERIOD 2020-2022.**

| AMAZONAS | ANCASH |
| --- | --- |
|  |  |
| APURIMAC | AREQUIPA |
|  |  |
| AYACUCHO | CAJAMARCA |
|  |  |
| CALLAO | CUSCO |
|  |  |
| HUANCAVELICA | HUÁNUCO |
|  |  |
| ICA | JUNÍN |
|  |  |
| LA LIBERTAD | LAMBAYEQUE |
|  |  |
| LIMA | LORETO |
|  |  |
| MADRE DE DIOS | MOQUEGUA |
|  |  |
| PASCO | PIURA |
|  |  |
| PUNO | SAN MARTIN |
|  |  |
| TACNA | TUMBES |
|  |  |
| UCAYALI | |
|  | |
